# Influence of Substrates on the Surface Characteristics and Membrane Proteome of *Fibrobacter succinogenes* S85

**DOI:** 10.1371/journal.pone.0141197

**Published:** 2015-10-22

**Authors:** Mahendra P. Raut, Esther Karunakaran, Joy Mukherjee, Catherine A. Biggs, Phillip C. Wright

**Affiliations:** The ChELSI Institute, Dept of Chemical and Biological Engineering, The University of Sheffield, Mappin Street, Sheffield, S1 3JD, United Kingdom; INRA Clermont-Ferrand Research Center, FRANCE

## Abstract

Although *Fibrobacter succinogenes* S85 is one of the most proficient cellulose degrading bacteria among all mesophilic organisms in the rumen of herbivores, the molecular mechanism behind cellulose degradation by this bacterium is not fully elucidated. Previous studies have indicated that cell surface proteins might play a role in adhesion to and subsequent degradation of cellulose in this bacterium. It has also been suggested that cellulose degradation machinery on the surface may be selectively expressed in response to the presence of cellulose. Based on the genome sequence, several models of cellulose degradation have been suggested. The aim of this study is to evaluate the role of the cell envelope proteins in adhesion to cellulose and to gain a better understanding of the subsequent cellulose degradation mechanism in this bacterium. Comparative analysis of the surface (exposed outer membrane) chemistry of the cells grown in glucose, acid-swollen cellulose and microcrystalline cellulose using physico-chemical characterisation techniques such as electrophoretic mobility analysis, microbial adhesion to hydrocarbons assay and Fourier transform infra-red spectroscopy, suggest that adhesion to cellulose is a consequence of an increase in protein display and a concomitant reduction in the cell surface polysaccharides in the presence of cellulose. In order to gain further understanding of the molecular mechanism of cellulose degradation in this bacterium, the cell envelope-associated proteins were enriched using affinity purification and identified by tandem mass spectrometry. In total, 185 cell envelope-associated proteins were confidently identified. Of these, 25 proteins are predicted to be involved in cellulose adhesion and degradation, and 43 proteins are involved in solute transport and energy generation. Our results supports the model that cellulose degradation in *F*. *succinogenes* occurs at the outer membrane with active transport of cellodextrins across for further metabolism of cellodextrins to glucose in the periplasmic space and inner cytoplasmic membrane.

## Introduction

Cellulose, an abundantly occurring organic polymer in the plant kingdom [1], has immense potential for the production of alternate fuels such as bioethanol [2]. Since cellulose is a highly stable polymer, expensive chemical hydrolysis is undertaken to ensure adequate yield of fuel from cellulose. Low cost production of fuel from cellulose necessitates the development of inexpensive pre-treatment techniques [2]. Enzymatic degradation of cellulose using microorganisms could be a promising low cost alternative to existing cellulose degradation strategies. However, lack of in-depth understanding of cellulose degrading organisms hinders the use of these microorganisms for cellulose degradation in consolidated biofuel generation processes.

There are many microorganisms capable of enzymatic degradation of cellulose, as reviewed by Lynd et al. [3]. The microbial consortia in the rumen of herbivores are well-specialised for cellulose degradation [4, 5]. *Fibrobacter succinogenes* S85 is a dominant cellulose degrading bacterium of the rumen community and actively degrades crystalline cellulose. However, unlike other cellulolytic microbes, it does not degrade cellulose by using a cellulosome or an extracellular free enzyme system [6]. The mechanism by which *F*. *succinogenes* degrades cellulose remains unknown.

Based on the genome sequence, several models have been proposed for cellulose degradation in *F*. *succinogenes* [7]. However, the lack of a systems level study precludes a full understanding of the mechanism of cellulose degradation in this bacterium. Preliminary studies on *F*. *succinogenes* suggest that: 1) adhesion is an essential pre-requisite to cellulose degradation and, 2) proteins may be involved in the adhesion process as protease treatments on whole cells abolish adhesion and subsequent cellulose degradation [8]. Indeed, a comparative study of membrane proteins from cells grown in glucose and cells grown in cellulose reveal about 16 outer membrane proteins were produced only when the cells were grown on cellulose. Furthermore, around 13 proteins with carbohydrate binding modules (CBM) were isolated from the cell membrane [8]. This suggests that the cellulose degradation machinery may be localised within the cell envelope in *F*. *succinogenes*. However, the presence of a high number of genes in the genome that were classified into 49 different families of carbohydrate active enzymes (glycoside hydrolases, carbohydrate binding modules (CBMs), carbohydrate esterases, and polysaccharide lyases) [7] suggests that a more rigorous investigation is necessary to understand the mechanism of cellulose degradation by *F*. *succinogenes*.

Moreover, in addition to proteins, cell surface polysaccharides, glycoproteins and lipoproteins have been discovered to play a major role in adhesion of heterotrophic bacteria to solid substrates [9]. Therefore, in order to better understand the mechanism of cellulose degradation by *F*. *succinogenes*, in which adhesion to cellulose is an important step, it is essential to: 1) consider the changes in net surface chemistry of *F*. *succinogenes* leading to adhesion in order to reassess the importance of proteins in the adhesion and cellulose degradation process, and 2) better understand the role of the abundant carbohydrate active enzymes proposed to be present in the *F*. *succinogenes* genome. In order to address the first objective of studying the comparative changes in the surface chemistry of *F*. *succinogenes* in the presence of cellulose when compared to glucose, we used surface characterisation techniques such as electrophoretic mobility analysis (EPM), the microbial adhesion to hydrocarbons (MATH) assay and Fourier transform infrared (FTIR) spectroscopy. These techniques have been previously used to study the changes in cell surface constituents of *Escherichia coli* and *Bacillus cereus* upon adhesion to a solid substrate [9, 10]. In order to address the second objective of better understanding the role of proteins in the adhesion to and degradation of cellulose, we employed a proteomics approach in which we selectively extracted the cell envelope proteins using biotin tags and identified the proteins by tandem mass spectrometry. Our results provide insight to better understand the mechanism of cellulose degradation by *F*. *succinogenes*. This is the overall aim of this paper.

## Materials and Methods

### Culture conditions and cultivation procedure

All chemicals and reagents were purchased from Sigma-Aldrich (Poole, UK) unless otherwise specified. The strain *F*. *succinogenes* S85 (ATCC19169) was kindly provided by Prof. Paul Weimer (US Dairy Forage Research Centre, Wisconsin, USA). *F*. *succinogenes* S85 was cultivated under anaerobic conditions at 38°C in modified Dehority medium (MDM) as described by Weimer et al. [11]. Culture media were prepared in triplicate with three different carbon substitutes; 1) 0.3% (w/v) glucose 2) 0.3% (w/v) microcrystalline (MC) cellulose and 3) 0.3% (w/v) acid swollen (AS) cellulose. The cultures were incubated anaerobically under CO_2_ at 38°C in 125 ml serum bottles (containing 100 ml medium), each fitted with a butyl stopper and an aluminium crimp seal. A starter culture was grown on glucose substrate for 18 hours at an optical density (OD) at wavelength 675 nm of ca. 0.42. One hundred millilitres of culture media was inoculated with 0.5 ml of starter culture. Specific growth rates of the bacterium under different substrate conditions were calculated from the growth measurement as absorbance (OD_675 nm_) versus time. Cells were harvested at the mid exponential phase, depending on the growth rate of the bacterial strain under different substrate conditions and processed further as per the protocol. For cultures grown on MC and AS cellulose, an additional step was performed, in which cells bound to residual cellulose were (S1 Fig) removed by centrifugation at 500xg for 5 min, before cell pellets were harvested by centrifugation at 8000xg for 10 min [8]. The cells bound to residual cellulose were detached from the cellulose particles using 0.1% methyl cellulose in buffer (M8) solution as described previously by Kudo et al. [11, 12] (detachment of cells confirmed by microscopy; see S2 Fig). Cells were harvested by centrifugation at 10000xg for 10 min and combined with previously harvested cells for further analysis. Since glucose is soluble in the medium, these additional steps were not applied to glucose grown cells.

AS cellulose was prepared by a method described elsewhere [13]. Briefly, 40 g of microcrystalline cellulose was mixed in 400 ml of 85% phosphoric acid solution and stored at 4°C for 30 min. The solution was then suspended in 3.6 l of pre-chilled deionised water and filtered. AS cellulose was then washed twice with 2.4 l of deionised water and resuspended in 2.4 l of pre-chilled distilled water and pH adjusted to 6.6 to 6.8. Finally, AS cellulose was washed twice and freeze dried.

### Transmission electron microscope specimen preparation and observation

The specimens were harvested at mid exponential phase for glucose and cellulose (AS and MC) conditions. The specimens were immediately fixed in 3% glutaraldehyde in 0.1% phosphate buffer and kept at 4°C overnight. The specimens were then washed with 0.1% phosphate buffer (twice with a 30 min interval) at 4°C. Secondary fixation was carried out in 2% aqueous osmium tetroxide for 2 hours at room temperature, followed by brief washing with distilled water and then 0.1% phosphate buffer. Dehydration was carried out through a graded series of ethanol (75%, 95% and 100%) steps for 30 min at room temperature and dried over anhydrous copper sulphate for 30 min. The specimens were then placed in an intermediate solvent, propylene oxide, for two changes of 30 min duration. Infiltration was accomplished by placing the specimens in a 50/50 mixture of propylene oxide/araldite resin. The specimens were left in this 50/50 mixture overnight at room temperature on a rotating mixer. The specimens were left in full strength Araldite resin (or similar) for 6–8 hours at room temperature after which they were embedded in fresh Araldite resin and cured for 48–72 hours at 60°C. Semi thin sections approximately 0.5 μm thick were cut on a Reichert Ultracut E ultramicrotome and stained with 1% toluidine blue in 1% borax for around 30 seconds on a hotplate or until the stain began to evaporate. The stain was differentiated in 50% alcohol for ca. 15–20 seconds, washed in water and dried on hotplate. It was subsequently mounted in DPX with a glass cover slip. Ultrathin sections (approximately 85 nm thick) were cut on a Reichert Ultracut E ultramicrotome on 200 mesh copper grids and stained for 30 min with 3% aqueous uranyl acetate followed by staining with Reynold’s lead citrate for 10 min. Sections were examined using a FEI Tecnai Transmission Electron Microscope (TEM) at an accelerating voltage of 80kV. Electron micrographs were taken using a Gatan digital camera.

### Carbohydrate determination

For glucose estimation, the culture broth was centrifuged at 8000xg for 10 min to remove bacterial cells and the supernatant was analysed by the Nelson-Somogyi method [14]. AS cellulose and MC cellulose was separated from cultures by centrifugation at 500xg for 5 min and was estimated using the method described by Updegraff [15].

### Characterisation of surface chemistry of *F*. *succinogenes*

#### Cell surface hydrophobicity

The microbial adhesion to hydrocarbon (MATH) was tested by the method described by Rosenberg et al. [16]. Briefly, bacterial cultures were harvested at the mid exponential phase, as described previously. Cells obtained by centrifugation at 8000xg for 10 min washed twice and resuspended in sterile 150 mM potassium chloride solution at pH 7. In this assay, a 150 mM solution of potassium chloride solution at pH 7 was used to minimise electrostatic effects, since these influence adhesion to n-hexadecane and subsequently may interfere with the results [17]. The cell density was adjusted to an OD of 1.0 at 675 nm. One millilitre of this suspension was transferred to new 2 ml Eppendorf tube and 200 μl of the solvent n-hexadecane was overlaid on each sample. The mixture was vortexed briefly and incubated at room temperature for 15 min. The mixture was vortexed again for 2 min and allowed to settle for 15 min at room temperature. Finally, the aqueous layer was carefully separated out and the OD at 675 nm was measured. The hydrophobicity index (HPBI) was calculated as follows [18].

$$\text{HPBI}=⟦\frac{\left(\text{A}1-\text{A}2\right)}{\text{A}1}⟧\times 100$$

Where A1 is the initial OD _675 nm_ before mixing with n-hexadecane and A2 is the OD _675 nm_ after mixing with n-hexadecane.

#### Electrophoretic mobility measurement

The electrophoretic mobility of cells over a pH range of 1.5 to 8 was measured to determine the cell surface charge. Cells obtained from glucose and cellulose substrate conditions were washed twice with 100 mM potassium chloride solution at pH 7 and OD _675 nm_ adjusted to 1.0. Twenty microlitres of the cell suspension was mixed with 1.8 ml of 100 ml KCl solution in a pH range of 1.5 to 8. The electrophoretic mobility of cells were analysed in a Zeta potential analyser (ZetaPALS, Brookhaven Instruments, UK). The measurement was conducted using an electric field of 2.5V cm at a frequency of 2.0Hz. The value reported for 3 biological replicates, is an average of 20 cycles with 6 runs conducted at 22°C. The isoelectric point of the bacterial cells was determined as the point of zero electrophoretic mobility of the cell from a pH vs electrophoretic mobility graph.

#### Functional group analysis by Fourier Transform Infrared (FTIR) Spectroscopy

The FTIR analysis was carried out as described elsewhere [19]. Intact cells obtained were washed thrice with 100 mM potassium chloride solution at pH 7. Cells were dissolved in same potassium chloride solution for further FTIR analysis.

FTIR analysis was carried out using a Fourier transform infrared spectrophotometer (IRprestige-21 Shimadzu Corporation, UK). Intact cells obtained were mounted on the spectrophotometer using a diamond Attenuated Total Reflectance (ATR) apparatus (Pike Technologies, USA). A blank spectrum without a biological sample was run as a background and the baseline shift of the spectra was corrected using the instrument’s software (IR solution). Spectra for samples were recorded in the range 600–3900 cm^-1^ using the Happ-Genzelapodisation over 64 scans with a resolution of 4 cm^-1^. Characteristic absorbance peaks of macromolecules of biological origin lie between wave numbers of 800 and 1800 [19,20]; thus an FTIR spectrum for this range only was considered for analysis. The spectral data processing was carried out using the IR solution software programme built into the Shimadzu FTIR instrument. Results obtained from the analysis were interpreted with previously published information [20, 21]. Principal component analysis (PCA) of the FTIR spectra was carried out with XLSTAT software (http://www.xlstat.com/; version 13.1.05) using the Pearson correlation.

### Cell envelope proteome analysis by biotinylation

#### Protein extraction

Biotinylation of *F*. *succinogenes S85* was performed as previously described [22] with some modification. Briefly, biological duplicates were used for each substrate condition. Two biological replicates have been successfully used for the proteomics analysis previously [23, 24]. Cells were harvested at mid exponential phase for glucose and cellulose grown cells. Cellulose grown cells were first separated from residual cellulose by centrifugation at 500xg for 2 min and further harvested by centrifugation at 8000xg for 5 min. The residual cellulose-bound cells were detached using 0.1% methylcellulose solution as suggested by Kudo et al. [12]. Cells were harvested by centrifugation and combined with previously harvested cells. Cell pellets were washed with phosphate-buffered saline (PBS) containing 1mM MgCl_2_ by centrifugation at 8000xg for 5 min at 4°C and pellets resuspended in the 1 ml PBS buffer. The final O.D at 675 nm was adjusted to a corresponding cell count of 2 x 10^9^ cells for all substrate conditions. Cells were further centrifuged and resuspended in 1 ml PBS containing 1mg EZ-LinkSulfo-NHS-SS-biotin (sulfosuccinimidyl-20 (biotinamido) ethyl-1,3-dithiopropionate) labels (Thermo, Pierce). The mixture was incubated at 4°C for 30 min and excess biotin was then quenched thrice by washing with 500 mM glycine-PBS solution. Biotin labelled cell pellets were resuspended in 1 ml of radioimmuno-precipitation assay buffer (RIPA) (25 mM Tris-HCl (pH 7.6), 150 mM NaCl, 1% NP-40, 1% sodium deoxycholate, 0.1% sodium dodecyl sulphate, 1.1000 dilution of protease inhibitor cocktail set II). Cell lysate was obtained by brief sonication (30 seconds sonication 1 min on ice; 2 cycles). Cell lysate was incubated on ice for 30 min with gentle occasional vortexing. At this stage, additional oxidised glutathione (100 M) was added to the lysate to protect disulphide bonds in the Sulfo-NHS-SS-biotin. The lysate was further centrifuged at 16000xg for 10 min at 4°C and the supernatant collected was stored at -80°C with 10% (v/v) glycerol until required for further analysis.

#### Neutravidin affinity purification of biotinylated proteins

Three hundred microlitres of neutravidin-agarose gel was washed three times with wash buffer containing 25 mM Tris-HCl (pH 7.6), 0.15 M NaCl, 0.5% NP40, 0.5% sodium deoxycholate, 0.05% SDS. The cell lysate was mixed with washed neutravidin-agarose gel and incubated on ice for 2 hours. The mixture was then centrifuged at 500xg for 1 min and supernatant was discarded. The gel slurry with biotinylated proteins was transferred to the column (Ultrafree-MC centrifugal filter device; Durapore polyvinylidene difluoride [PVDF], 5.0 m pore size;millipore). Unbound proteins were removed by washing with a washing buffer (25 mM Tris-HCl [pH 7.6], 0.65 M NaCl, 0.1% NP40) twice, followed by washing with buffer (25 mM Tris-HCl [pH7.6], 1.15 M NaCl, 0.1% NP-40) and finally with Tris buffer (25 mM Tris-HCl [pH 7.6], 0.15 M NaCl) at 200xg for 15–20 seconds. Gel bound proteins were eluted thrice with 5% 2-mercaptoethanol-PBS at 30°C for 30 min. Proteins were precipitated by 10% trichloroacetic acid (TCA) and centrifuged at 18000xg for 10 min at 4°C [25]. The protein pellets were finally washed with ice cold acetone and air dried. The purified proteins then re-dissolved in 0.5 M triethylammonium bicarbonate (TEAB) buffer containing 0.1% RapiGest (protein solubilising reagent) and further proteomics analysis was carried out.

#### In-gel digestion for protein identification and peptide recovery

SDS-PAGE was performed on neutravidin-agarose affinity purified proteins separated via the standard procedure described elsewhere [26]. In-gel digestion of proteins was achieved as previously described by Karunakaran et al. [19]. Briefly, protein bands obtained by 1-D gel electrophoresis were sliced into 10 pieces and destained twice with 200 l of 200 mM ammonium carbonate (AB) in 40% acetonitrile (ACN) by incubating at 37°C for 30 min. The supernatant was discarded and gel pieces dried in a vacuum concentrator. Proteins entrapped in the gel were reduced and alkylated using 200 l of 10 mM dithiothreitol (DTT) by incubating at 56°C for 1 hour and 200 l of 55 mM iodoacetamide (IAA) at room temperature for 30 min in the dark respectively. Gel pieces were washed twice with 200 l of 50 mM AB solution for 15 min at room temperature followed by 200 l of 50 mM AB in 50% ACN for 15 min at 37°C. Subsequently, samples were digested with 1:50 (w/w) trypsin (Applied Biosystems, USA) containing 0.1% RapiGest (protein solubilising agent) and 50 l of 40 mM AB in 9% ACN for approximately 16 hours by incubation at 37°C. After incubation, the samples were centrifuged at 13000xg for 10 seconds and the supernatant was collected in a new Eppendorf tube. Peptides were extracted twice with 50 l of 5% formic acid (FA) and 50 l of 100% ACN. Finally, all the liquid extracted was combined and peptides were vacuum dried (Vacuum concentrator 5301, Eppendorf, UK) and stored at -20°C until further analysis by LC-MS/MS.

#### ESI mass spectrometry and identification of proteins

Peptides obtained from in-gel digestion were resuspended in reverse phase transfer buffer (3% acetonitrile and 0.1% FA) and submitted to a QStarXL Hybrid ESI-qQ-TOF-MS/MS (AB SCIEX, Concord, Ontario Canada) coupled with an online capillary liquid chromatography system (Ultimate 3000, Dionex, Surrey UK). Ten microliters of each peptide sample was injected into the nanoLC-ESI-MS/MS system and then separation performed by a PepMap C-18 RP capillary column (LC Packings) with a constant flow rate of 300 nl min^-1^. The buffers used for the liquid chromatography were Buffer A_ms_ (3% ACN with 0.1% FA), and Buffer B_ms_ (97% ACN with 0.1% FA), and the gradient was as follows: 0% Buffer B_ms_ for 3 min, 3% to 36% Buffer B_ms_ for 90 min, 36% to 90% of Buffer B_ms_ for 2 min, 90% of Buffer B_ms_ for 6 min, 3% of buffer B_ms_ for 13 min. Two precursors of charge +2 and+3 (intensity binning) for each TOF-MS scan (350–1200 *m/z*) were dynamically selected and isolated for MS/MS fragment ion scans (65–1600 *m/z*).

#### Peptide identification

Data obtained from tandem MS analysis were converted to Mascot generic files (MGF) using Data-Analysis software ver. 4.0 (Bruker Daltonics, Coventry UK). Converted peak lists were then submitted to an in-house software Phenyx algorithm cluster (Binary version 2.6; Genebio, Geneva) for peptide identification. The search was performed against the UniProt database for *F*. *succinogenes* S85 (taxon ID 59374) containing 3815 protein sequences downloaded from Uniprot (October 2013). Simultaneously, the search was performed against a reverse database based on the target database of *F*. *succinogenes*. Search parameters were set at a mass tolerance of 0.4 Da and MS/MS tolerance of 0.4 Da. Peptide level filters were set to a z-score of 5.0, max *p-*value significance of 1.0E-5. AC score was set at 5. The search space was also limited by trypsin peptides with a maximum of 1 missed cleavage. The results of the search against the target and reverse database was used to calculate the false discovery rate. Stringent parameters for protein identifications, across all three conditions and replicates, were used with a minimum of two unique peptides and a false discovery rate below 1.5% at the protein level. The semi-quantitative comparisons of protein concentrations between the treatments were considered based on number of confidently observed peptide identifications.

## Results

### Bacterial growth and substrate consumption

**Fig 1:**
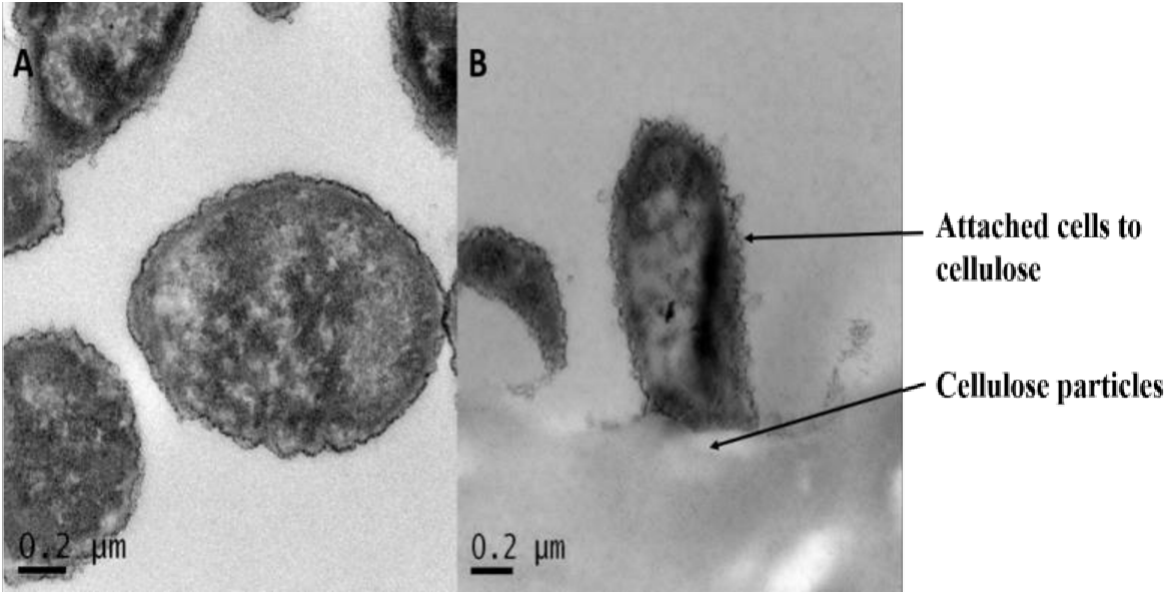
Transmission electron microscopy (TEM) images of the bacterium *F*. *succinogenes* S85 cells grown on glucose (A) and cellulose substrate (B).

In this study, *F*. *succinogenes* was grown on three different substrates, glucose, acid swollen (AS cellulose) and microcrystalline cellulose (MC cellulose). Adhesion of the cells to cellulose was observed (Fig 1), which is in agreement with earlier studies [27].

**Fig 2:**
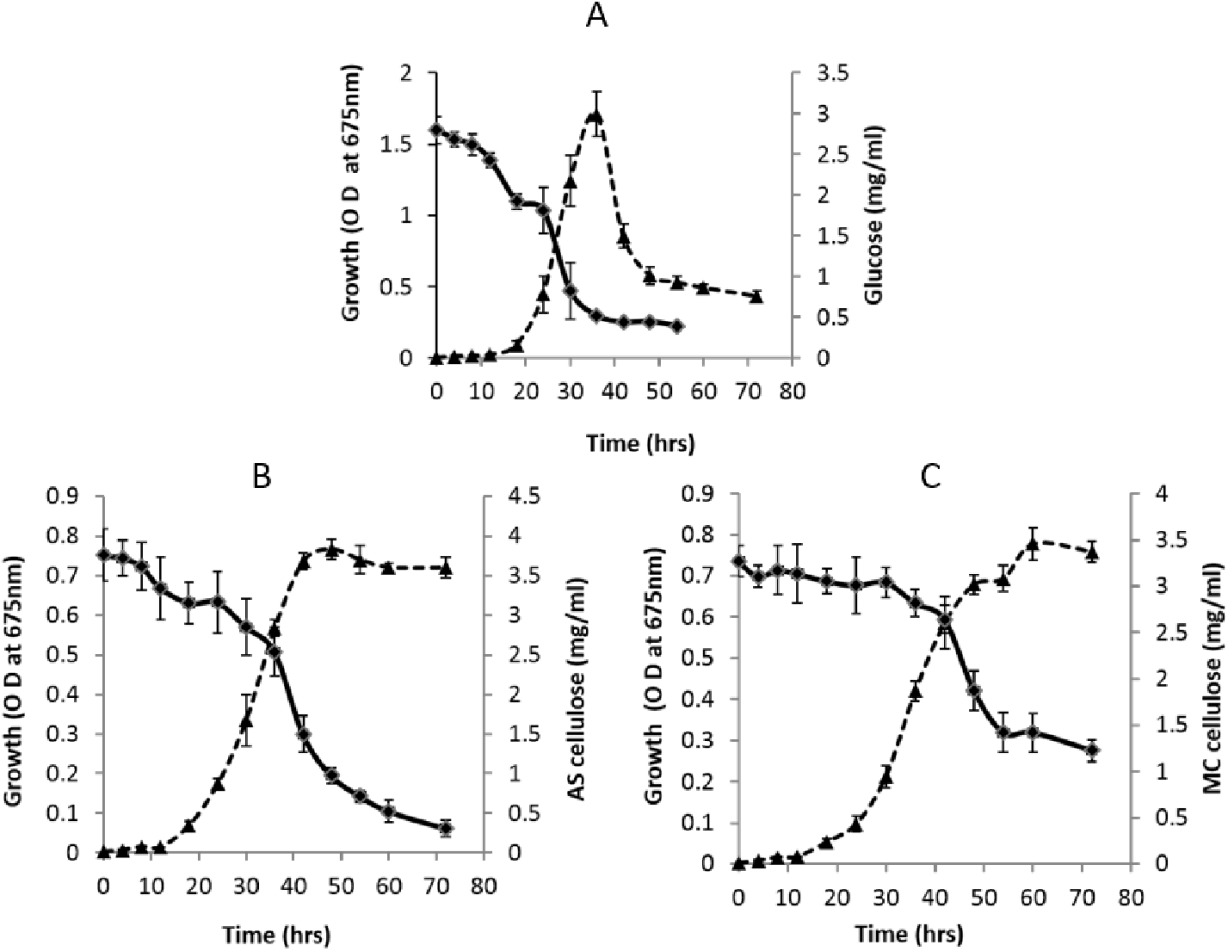
Growth and substrate consumption profile of *F*. *succinogenes* S85: A) Glucose B) Acid swollen (AS) cellulose ♦ and C) Microcrystalline (MC) cellulose (represents substrate utilisation and ▲ represents OD_675_).

Before carrying out optical density measurements for the measurement of growth rate and for subsequent analyses in this study, the cells grown on cellulose were detached from cellulose via methylcellulose treatment. The substrate consumption profile and growth rate of *F*. *succinogenes* S85 is shown in Fig 2.

*F*. *succinogenes* S85 grew on glucose, AS cellulose and MC cellulose with growth rates of 0.20, 0.098 and 0.084 h^-1^ respectively. A corresponding decrease in the concentration of substrates was seen. The rate of substrate consumption rate was calculated from the linear portion of the graph during the log phase. Glucose was consumed at a rate of 0.130 mg/ml/hr, whilst AS cellulose and MC cellulose were consumed at a rate of 0.071 mg/ml/hr, and 0.057 mg/ml/hr respectively.

Results indicate that *F*. *succinogenes* S85 grew faster in the presence of glucose compared to cellulose substrates. However, the cells grown on glucose did not achieve a sustained stationary phase, as seen with cells grown on cellulose substrates. This result is in agreement with previous studies, which noted that either glucose depleted conditions or nitrogen depletion, causes the cells to produce extracellular proteases, which result in autolysis of the cells [26, 28, 29]. In contrast, cells grown on cellulose substrates were characterized by an extended log phase, followed by a more sustained stationary phase. For the subsequent experiments, the cells were harvested at the mid-exponential phase of growth in all substrate conditions.

### Hydrophobicity and Surface charge

Changes in bacterial cell surface chemistry can be detected as changes in the net hydrophobicity and charge of the cell surface. The MATH assay is routinely used to measure the extent of hydrophobicity of bacterial cell surfaces [10, 19]. A removal of less than 30% of the cell suspension from the aqueous phase into the organic phase implies that the cell surface is hydrophilic [30, 31].

Our results suggest that the surface of cells grown on glucose is hydrophilic in nature (14.9% removal into organic phase). However, on exposure to the different cellulose substrates, the cell surface becomes more hydrophilic compared to the surface of glucose grown cells (11.1% and 11% for AS and MC cellulose respectively). The student t-test performed among the treatments shows significant differences between glucose and the two types of cellulose treatments (*p* value = 0.0464 & 0.0409, respectively) at 95% confidence, whereas no significance difference was observed between the two cellulose treatments (*p* value = 0.484).

The zeta potential (a function of the surface charge) of a cell can be calculated from the measurement of the electrophoretic mobility (EPM) in an applied external electric field. The direction and rate of the cell mobility depends on polarity, net surface charge, temperature, ionic strength and pH of the medium.

**Fig 3:**
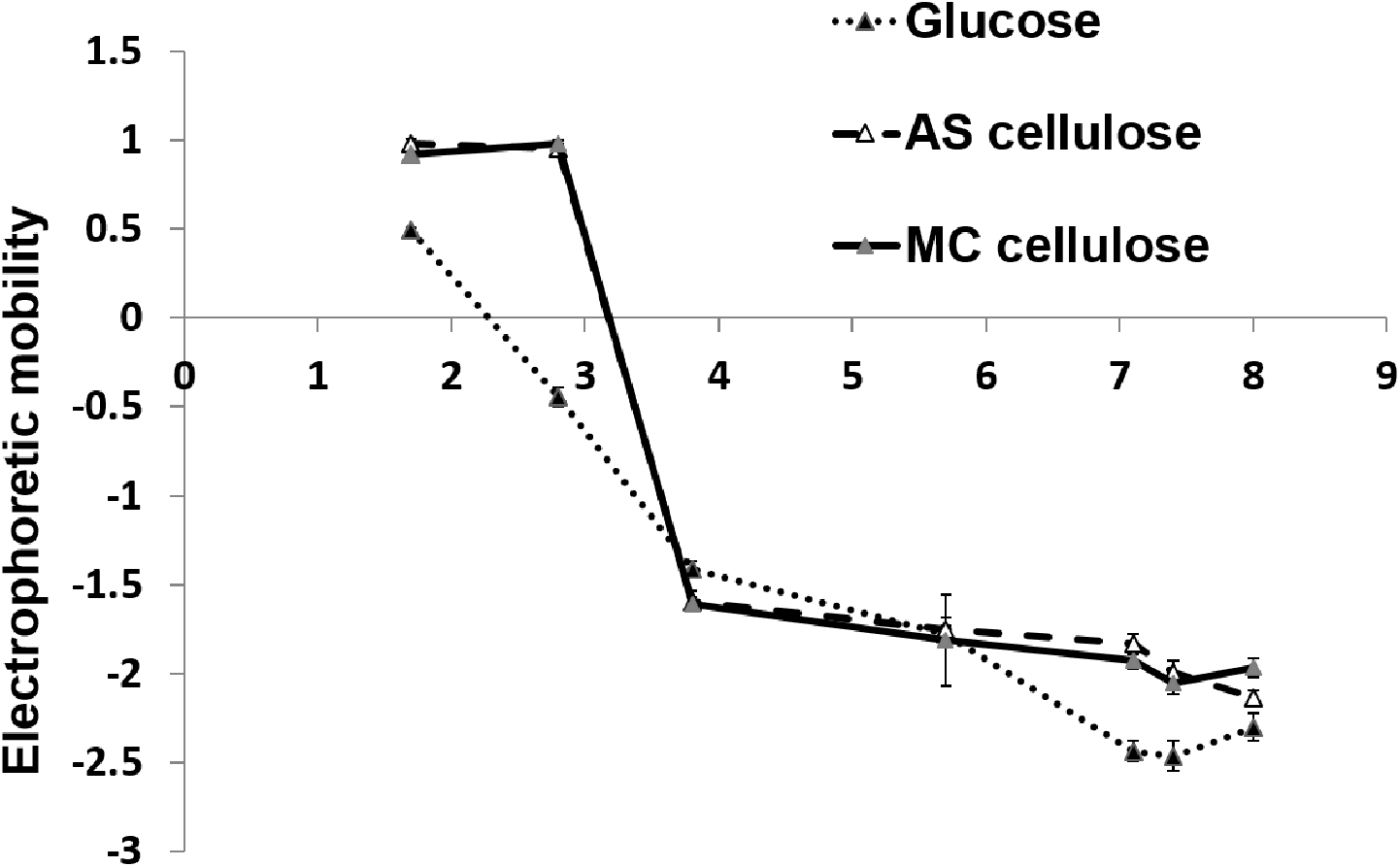
Electrophoretic mobility of *F*. *succinogenes* S85 cells under different carbon substrate conditions as a function of pH. Error bars = SE value.

The EPM data was plotted as a function of pH (Fig 3). The EPM of cells grown in either glucose or cellulose becomes more negative as the pH of the environment becomes more basic. This trend has previously been observed in bacteria such as *Escherichia coli*, *Bacillus cereus*, *Bacillus brevis*, *Staphylococcus aureus*, *Pseudomonas putida* and *Alcaligenes faecalis* [19, 32–35] and arises due to the protonation/deprotonation of surface exposed functional groups based on the pH of the medium.

Although the EPM of cellulose grown cells at pH 7 is only marginally less electronegative than the glucose grown cells, significant differences can be seen in the isoelectric points of the cells grown with different substrates. The isoelectric point is the pH at which the net EPM of the cell is zero [36]. The isoelectric point for cells grown in glucose was obtained at pH 2.2, whereas, for cells grown with AS cellulose and MC cellulose, the isoelectric points were between pH 3 and 3.5. Such a shift in the isoelectric point to a less acidic pH can only be observed if there is an increase in protein associated ammonium (R-NH_3_^+^/ R-NH_2_) on the cell surface [10].

### Fourier Transform Infra-Red Spectroscopy

**Fig 4:**
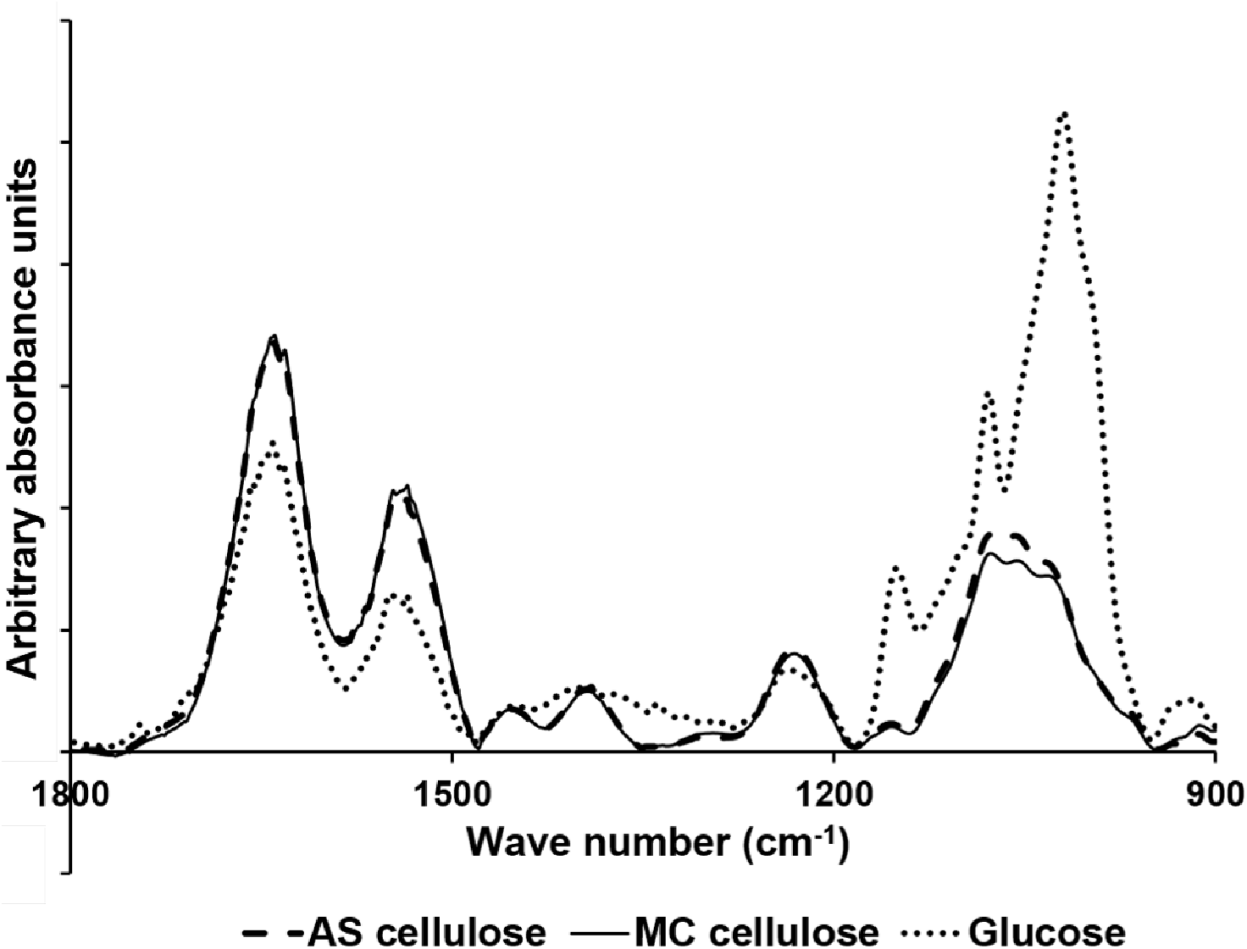
Comparative FTIR spectrum of *F*. *succinogenes* S85 strains grown under different carbon substrate conditions.

The FTIR spectrum of whole cells is a combination of the unique spectral fingerprints of individual biochemical components such as proteins, lipids, and carbohydrates [21]. The FTIR spectrum was recorded between 600 cm^-1^ to 4000 cm^-1^. However, the most useful information can be obtained from the spectral region between 800 cm^-1^ and 1800 cm^-1^ [19]. Therefore, the FTIR spectrum was considered for bacterial surface analysis within this range. The ATR-FTIR spectra of the cells grown on glucose and cellulose substrates are presented in Fig 4.

A comparison of the FTIR spectra shows that major differences are exhibited in the ring vibrations of C-O-(C/P) and C-O group of polysaccharides, (1200–900 cm^-1^) and the C-O-C group of esters (1230 cm^-1^) [20]. Considerable differences are also observed in the amide I (C = O) and amide II (N-H) regions that lie between 1700 cm^-1^ and 1500 cm^-1^ [20]. The results suggest a decrease in the cell surface polysaccharide display and a concomitant increase in the cell surface protein display when the cells are grown in cellulose versus glucose grown cells.

**Fig 5:**
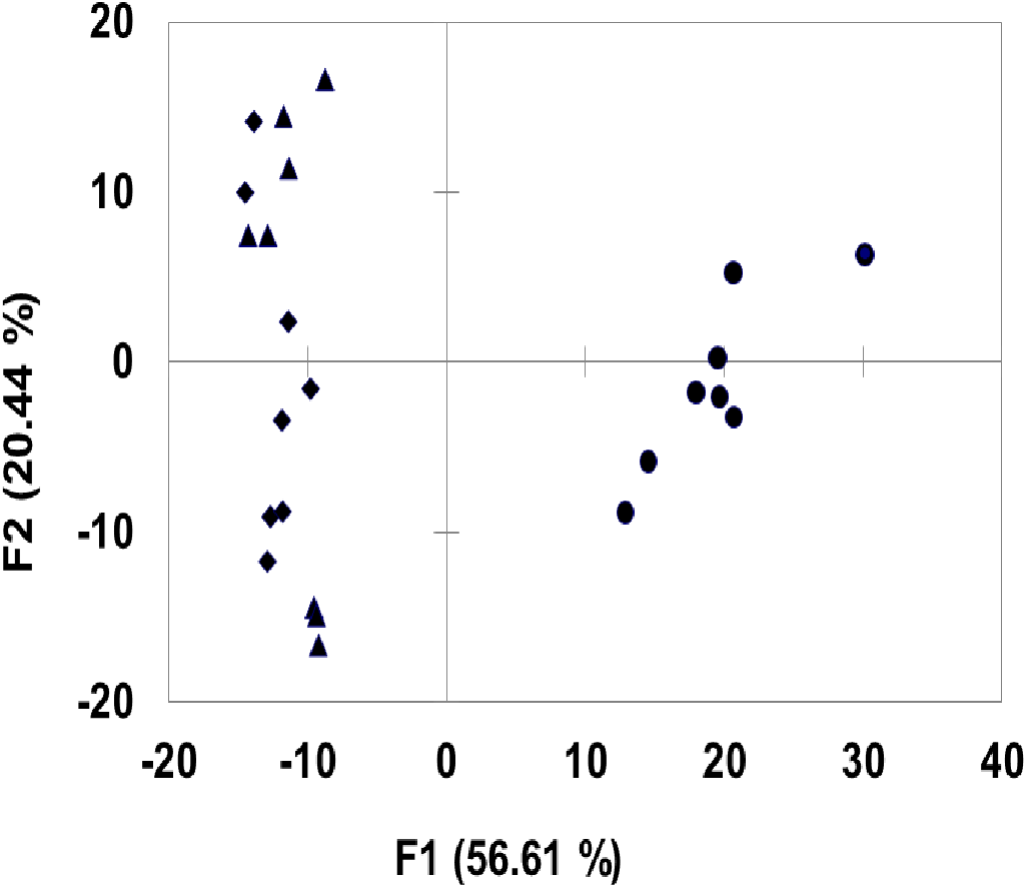
Principal component analysis (PCA) of ATR-FTIR spectra of *F*. *succinogenes* S85 cells grown on (●) Glucose, (▲) AS cellulose, (♦) MC cellulose.

Furthermore, principal component analysis (PCA) of the different spectra of cells grown on glucose and cellulose substrates was carried out. The PCA analysis (Fig 5) reinforces the fact that the cell surface of cellulose grown cells is distinctly different from that of glucose grown cells. No significant difference was seen in the surface of the cells grown in the presence of the two different forms of cellulose.

### Identification of cell envelope proteins

Biotinylated cell envelope-associated proteins were enriched by neutravidin affinity purification, as described in the methods section. To evaluate the enrichment procedure, a control experiment was carried out using proteins prepared from unbiotinylated *F*. *succinogenes* cells grown on glucose (S3 Fig). The absence of proteins in the SDS-PAGE gel of the control sample clearly demonstrated that the proteins found in the SDS-PAGE gel of the biotinylated enriched samples did not arise due to inadequate wash steps or non-specific binding of proteins to neutravidin. In order to identify the proteins found in the biotinylated enriched samples, an in-gel trypsin digestion workflow followed by tandem mass spectrometry was carried out.

**Fig 6:**
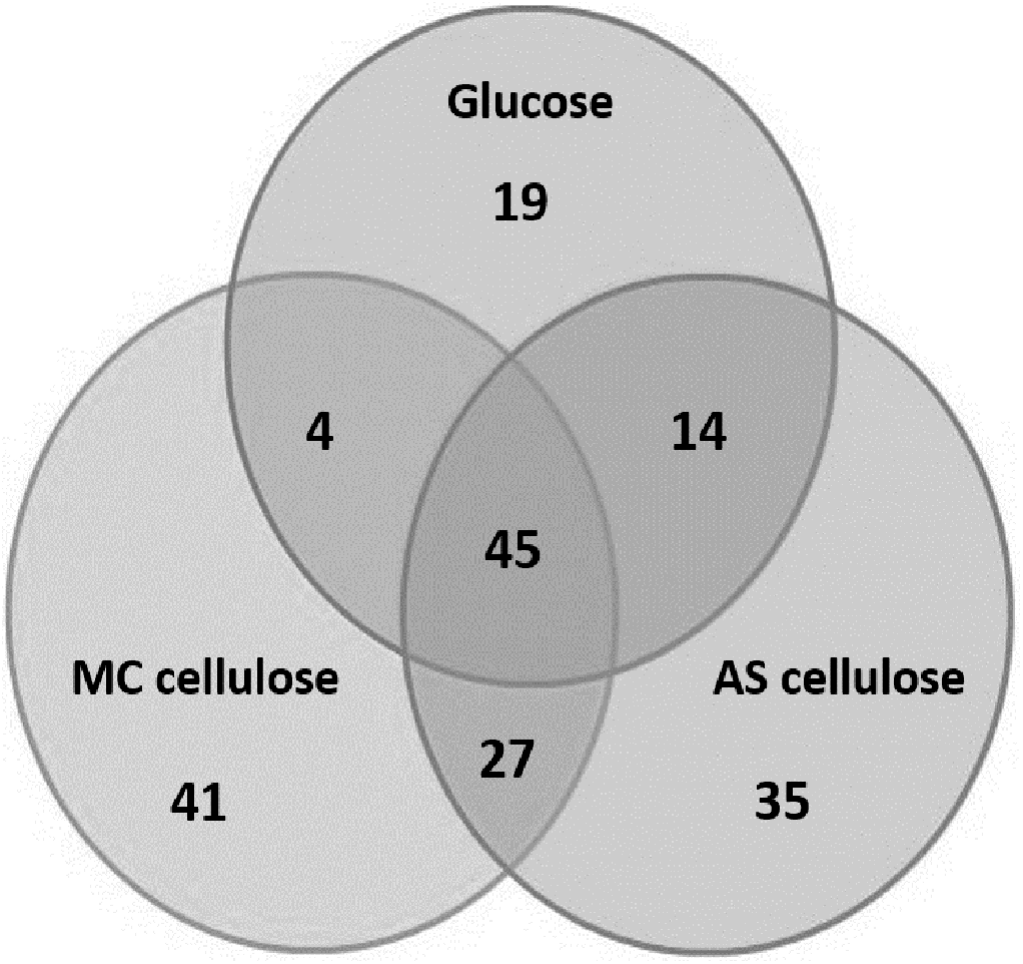
Venn diagram showing distribution of the 185 cell envelope-associated proteins among three different substrate conditions.

Across the three substrate conditions, a total of 347 proteins were identified with at least 2 unique peptides. Of these, 185 proteins were classified as non-cytoplasmic proteins, whilst 162 proteins were classified as cytoplasmic proteins by PSORTb [37]. The identified cytoplasmic proteins have been excluded from further discussion, but are provided in S1 Table. The distribution of the 185 cell envelope proteins across the three substrate conditions is summarised in Fig 6. The sequence, charge and score of all identified peptides are given in S3 Table.

Based on their role in metabolic processes, the 185 cell envelope proteins identified can be further divided into three categories: 1) predicted to be involved in cellulose degradation (Table 1; 25 proteins), 2) energy generation, transport and protein-protein interaction (Table 2; 43 proteins) and 3) putative uncharacterised lipoprotein/membrane proteins (S2 Table; 117 proteins).

**Table 1:** List of predicted cell envelope proteins associated with cellulose degradation in *F*. *succinogenes* S85.

Locus ID	Protein description	G*	MC*	AS*	Family^a^	Location^b^	Gravy Index^c^	Molecular mass (kDa)^c^	pI^c^	Presence of signal peptide (amino acid position)^d^	Ref
**Proteins common to all substrates**											
Fisuc_1979 FSU_2502	Fibro-slime domain protein	2	17	12	-	Non cytoplasmic	-0.312	169.38	5	Yes (32–34)	[8]
Fisuc_1802	Glycoside hydrolase family 8	3	7	8	GH8	Non cytoplasmic	-0.24	79.81	5.63	No	[7,41]
Fisuc_3111 FSU_0382	Carbohydrate binding family 11 (Cellulase)	9	3	6	CBM30, CBM11, GH51	Non cytoplasmic	-0.551	118.616	7.81	Yes (23–24)	[7,42]
Fisuc_1525 FSU_2007	Cellulose-binding domain protein	2	2	3	CBM30	Non cytoplasmic	-0.301	29.2	6.46	Yes (35–36)	[7,8]
Fisuc_1230 FSU_1691	Extracellular solute-binding protein family 5	9	20	10	-	Unknown	-0.336	67.554	5.69	Yes (21–22)	
**Proteins found only in cellulose treatments**											
Fisuc_1932 FSU_2442	Alpha amylase catalytic region	-	-	2	GH13	Unknown	-0.564	66.93	6.25	No	-
FSU_2932	Cadherin domain protein	-	2	-	-	Outer membrane	-0.422	317.91	4.69	Yes (23–24)	-
Fisuc_2900 FSU_0162	Cellodextrin-phosphorylase	-	8	5	GH94	Cytoplasmic membrane	-0.352	93.662	8.16	No	-
Fisuc_1224 FSU_1685	Cellulase	-	3	6	GH5	Extracellular	-0.278	80.027	7.55	Yes (20–21)	[7]
Fisuc_1523 FSU_2005	Cellulase (Glycoside hydrolase family 5)	-	-	2	GH5	Unknown	-0.239	42.062	4.94	Yes (19–20)	[7]
Fisuc_2704 FSU_3272	Conserved domain protein (Glucosylceramidase)	-	-	7	GH116	Unknown	-0.216	116	6.23	No	-
FSU_2361	Endoglucanase	-	-	2	GH9	Non cytoplasmic	-0.248	67.365	6.06	Yes (26–27)	[7,43]
Fisuc_2230 FSU_2772	Endoglucanase 3 (Cellulase 3)	-	3	3	GH5 CBM11	Non cytoplasmic	-0.359	73.424	4.61	Yes (25–26)	[7,44]
Fisuc_1465 FSU_1938	Extracellular ligand-binding receptor	-	3	4	-	Non cytoplasmic	-0.212	67.21	9.25	Yes (19–20)	-
FSU_1047	Extracellular solute-binding protein	-	5	-	-	Unknown	-0.218	57.575	7.77	No	-
Fisuc_2019 FSU_2542	Extracellular solute-binding protein family 5	-	-	2	-	Periplasmic	-0.46	70.02	6.33	Yes (24–25)	-
Fisuc_2249 FSU_2794	Fibrobacter succinogenes major paralogous domain protein	-	3	10	-	Non cytoplasmic	-0.387	70.12	4.57	No	-
Fisuc_1219	Glycoside hydrolase family 8	-	-	4	GH8	Non cytoplasmic	-0.037	52.666	4.93	Yes (25–26)	[7]
Fisuc_0393 FSU_0809	Glycoside hydrolase family 9	-	3	2	CBM1, GH9	Non cytoplasmic	-0.335	233.01	4.97	Yes (18–19)	[7]
Fisuc_1252 FSU_1715	Peptidoglycan glycosyltransferase	-	12	2	GT51	Unknown	-0.35	126.864	6.76	No	-
Fisuc_1192 FSU_1653	Periplasmic solute binding protein	-	-	3	-	Cytoplasmic membrane	-0.125	36.391	5.03	Yes (20–21)-	-
**Other proteins**											
Fisuc_2377 FSU_2931	Cadherin (Cadherin domain protein)	4	-	2	-	Non cytoplasmic	-0.456	22.06	4.69	Yes (27–28)	-
Fisuc_2503 FSU_3071	Extracellular solute-binding protein family 3	3	-	2	-	Periplasmic	-0.132	28.75	5.47	Yes (21–22)	-
Fisuc_0377	Fibro-slime family protein	3	-	-	-	Non cytoplasmic	-0.433	98.69	5.16	Yes (21–22)	-
FSU_3194	Fibronectin type III domain protein	2	-	-	-	Unknown	-0.607	77.87	4.78	No	-

* G–glucose; MC–microcrystalline cellulose; AS–acid swollen cellulose. The numbers under these columns represent the number of unique valid peptide sequences on which protein identification is based.

^a^Carbohydrate active enzymes database (http://www.cazy.org/) [40]

^b^Location of the given proteins predicted by the PSORTb subcellular localization prediction tool version 3.0 [37]

^c^Theoretical isoelectric point, molecular mass and gravy index of the given protein, as predicted by the ExPASy Compute pI/MW tool [38]

^d^Determined by SignalP v.3.0 [39] the numbers in parentheses indicates the amino acids between which cleavage is predicted to occur in the given protein

**Table 2:** List of predicted cell envelope proteins associated with ion conductivity, transportation, signal transduction and protein-protein interaction in *F*. *succinogenes* S85.

Locus ID	Protein description	G*	MC*	AS*	Location^b^	Gravy Index^c^	Molecular mass (kDa)^c^	pI^c^	Presence of signal peptide (amino acid position)^d^	Ref
**Proteins present in all conditions**										
Fisuc_1592	OmpA/MotB domain protein	25	20	25	Outer membrane	-0.386	73.841	4.72	Yes (17–18)	-
FSU_2398	TPR domain protein	7	18	20	Non cytoplasmic	-0.617	146.67	8.5	Yes (20–21)	[8]
Fisuc_3112 FSU_0383	DSBA oxidoreductase	7	13	13	Non cytoplasmic	-0.413	27.626	8.58	Yes (24–25)	-
Fisuc_2509 FSU_3077	OmpA family protein	7	8	11	Outer membrane	-0.381	83.917	4.75	Yes (19–20)	-
Fisuc_1892 FSU_2397	TPR domain protein	4	9	7	Non cytoplasmic	-0.341	83.84	5.51	Yes (23–24)	[8]
Fisuc_2917 FSU_0180	OmpA family protein	5	2	6	Outer membrane	-0.272	70.79	5.27	Yes (17–18)	-
Fisuc_0369	WD40 domain protein beta Propeller	4	4	5	Non cytoplasmic	-0.373	44.076	7.74	Yes (21–22)	-
Fisuc_0289 FSU_0701	Efflux transporter, RND family, MFP subunit	5	5	4	Non cytoplasmic	-0.115	37.256	9.45	No	-
Fisuc_0978	Capsular exopolysaccharide family	2	8	4	Cytoplasmic membrane	-0.242	77.96	8.94	No	-
Fisuc_1591 FSU_2077	Capsular exopolysaccharide family	4	3	4	Cytoplasmic membrane	-0.092	78.322	8.14	No	-
Fisuc_0299 FSU_0711	Tetratricopeptide repeat protein	2	4	3	Periplasm	-0.656	49.015	9.14	Yes (29–30)	-
Fisuc_2987 FSU_0252	Ankyrin	2	3	3	Non cytoplasmic	-0.252	26.75	8.97	Yes (19–20)	-
Fisuc_1894 FSU_2400	MotA/TolQ/ExbB proton channel	4	4	2	Cytoplasmic membrane	0.461	23.06	9.14	No	-
Fisuc_2152	Outer membrane assembly lipoprotein YfiO	2	2	2	Unknown	-0.707	34.31	8.63	Yes (32–33)	-
Fisuc_0858 FSU_1302	Protein-export membrane protein SecD	6	4	2	Cytoplasmic membrane	0.285	93.186	8.96	No	-
**Proteins found only in cellulose**										
Fisuc_2892	OmpA/MotB domain protein	-	2	6	Outer membrane	-0.36	32.21	5.53	Yes (19–20)	-
Fisuc_1897 FSU_2403	TonB family protein	-	8	3	Unknown	-0.393	32.452	9.84	No	-
Fisuc_1226 FSU_1687	ABC transporter related protein	-	4	3	Cytoplasmic membrane	-0.323	30.503	8.6	No	-
Fisuc_1895 FSU_2401	Biopolymer transport protein ExbD/TolR	-	3	2	Unknown	-0.2	32.44	4.72	No	-
Fisuc_0149 FSU_0552	Sulfate ABC transporter, periplasmicsulfate-binding protein	-	-	2	Periplasm	-0.401	37.761	5.68	Yes (22–23)	-
Fisuc_2367 FSU_2921	Preproteintranslocase, SecG subunit	-	2	2	cytoplasmic membrane		16.96	8.73	No	-
Fisuc_0288 FSU_0700	Outer membrane efflux protein	-	2	-	Outer membrane	-0.378	52.346	5.31	Yes (21–22)	[8]
FSU_2396	OmpA family protein	-	11	-	Outer membrane	-0.408	55.97	4.86	Yes (28–29)	[8]
Fisuc_1896 FSU_2402	Biopolymer transport protein ExbD/TolR	-	2	-	Unknown	0.2	18.24	4.69	No	-
Fisuc_0038 FSU_0431	TPR repeat-containing protein	-	3	-	Non cytoplasmic	-0.384	39.988	6.22	No	-
Fisuc_0042 FSU_0435	MotA/TolQ/ExbB proton channel	-	7	-	Non cytoplasmic	-0.079	57.895	9.54	Yes (49–50)	-
Fisuc_2074 FSU_2602	Large-conductance mechanosensitive channel	-	2	-	Cytoplasmic membrane	0.462	15.94	9.21	No	-
Fisuc_0201	MotA/TolQ/ExbB proton channel	-	2	-	Cytoplasmic membrane	0.319	24.61	7.78	No	-
Fisuc_0743 FSU_1181	ABC transporter related protein	-	2	-	Cytoplasmic membrane	-0.071	29.041	5.61	No	-
Fisuc_0884 FSU_1330	Secretion protein HlyD family protein	-	3	-	Cytoplasmic membrane	-0.189	36.175	8.8	No	-
Fisuc_0885 FSU_1331	Outer membrane efflux protein	-	6	-	Outer membrane	-0.345	61.621	5.22	Yes (19–20)	-
Fisuc_2839 FSU_0095	V-type ATPase, D subunit	-	2	-	Unknown	-0.448	23.981	9.87	No	-
Fisuc_3033 FSU_0298	Mechanosensitive ion channel family protein	-	3	-	Cytoplasmic membrane	0.55	29.387	6.32	No	-
Fisuc_1227 FSU_1688	Oligopeptide/dipeptide ABC transporter, ATP-binding protein	-	5	-	Cytoplasmic membrane	-0.138	36.622	8.33	No	-
Fisuc_1395 FSU_1863	Capsular polysaccharide biosynthesis domain protein	-	3	-	Unknown	-0.002	43.793	5.5	Yes (17–18)	-
Fisuc_1571 FSU_2056	Outer membrane efflux protein	-	2	-	Outer membrane	-0.387	47.008	5.45	Yes (20–21)	-
Fisuc_1658 FSU_2147	TPR repeat-containing protein	-	4	-	Non cytoplasmic	-0.676	27.923	7.61	Yes (22–23)	-
Fisuc_0457 FSU_0874	Band 7 protein	-	4	-	Cytoplasmic membrane	-0.322	55.171	5.4	No	-
FSU_0746	Pentapeptide repeat domain protein	-	10		Extracellular	-0.396	48.123	9	Yes (25–26)	-
**Other proteins**										
Fisuc_1891	OmpA/MotB domain protein	7	-	11	Unknown	-0.458	53.24	4.74	No	-
Fisuc_0331	Pentapeptide repeat protein	13		9	Extracellular	-0.401	47.89	9	Yes (23–24)	-
FSU_0151	OmpA family protein	2	-	-	Cytoplasmic membrane	-0.566	23.937	6.65	No	-
Fisuc_1316 FSU_1783	PEGA protein	4	-	-	Non cytoplasmic	-0.451	20.24	8.83	Yes (20–21)	[8]

* G–glucose; MC–microcrystalline cellulose; AS–acid swollen cellulose. The numbers under these columns represent the number of unique valid peptide sequences on which protein identification is based.

^b^Location of the given proteins predicted by the PSORTb subcellular localization prediction tool version 3.0 [37]

^c^Theoretical isoelectric point, molecular mass and gravy index of the given protein, as predicted by the ExPASy Compute pI/MW tool [38]

^d^Determined by SignalP v.3.0 [39] the numbers in parentheses indicates the amino acids between which cleavage is predicted to occur in the given protein

The 25 proteins predicted to be associated with cellulose degradation (Table 1) were classified into different families according to the CAZy database (http://www.cazy.org) [40] and include: 11 glycoside hydrolases (GH) and a glycosyltransferase family GT51. Some of these enzymes have carbohydrate-binding modules (CBM) including CBM1, CBM11 and CBM30, and are therefore divided into multiple CAZy family memberships. Other identified proteins, predicted to be associated with cellulose degradation, belong to; fibro-slime family proteins, fibronectin type III domain proteins, cadherin and extracellular solute binding proteins. Of these, only 10 proteins have been previously identified [8, 41–44].

Of the 25 proteins associated with cellulose degradation, 5 proteins were found to be present irrespective of whether the cells were grown in the presence of glucose or cellulose. These include the cellulose binding domain protein family (Fisuc_1525), cellulase (Fisuc_3111), GH8 family protein (Fisuc_1802), extracellular solute binding protein (Fisuc_1230) and fibroslime family protein (Fisuc_1979). Fourteen proteins were present exclusively during cellulose treatments, which strongly suggest their involvement in cellulose adhesion and degradation.

The 43 proteins identified and predicted to play a role in energy generation, transport and protein-protein interaction are given in Table 2. These proteins are involved in various outer membrane-associated processes including OmpA family proteins, TonB family proteins, TPR domain proteins, substrate transporter proteins, efflux transporter proteins, proton channel proteins and capsular/surface repeat proteins.

In addition, 117 other proteins were identified, including mostly putative/uncharacterised proteins; these are provided in the S2 Table. Identification of such a large number of proteins with an unknown function is unsurprising, since 50% of the open reading frames (ORF) identified during genomic annotation have unknown functions in *F*. *succinogenes* S85 [8].

## Discussion

Previous studies [7, 8] have suggested that cellulose degradation in *F*. *succinogenes* is a cell envelope-associated process, which includes adhesion to cellulose as a pre-requisite step. In this study, TEM images demonstrated that adhesion of *F*. *succinogenes* to cellulose occurs during cellulose degradation. Preliminary experiments have suggested that cell envelope proteins play an important role in both the adhesion process, as well as the subsequent degradation of cellulose. However, increasing evidence that adhesion of heterotrophic bacteria to abiotic substrates is mediated by a complex milieu of polymers, including proteins, necessitated a re-assessment of the role of proteins in the adhesion of *F*. *succinogenes* to cellulose [45]. In addition, previous studies on outer membrane proteins of *F*. *succinogenes* have indicated that the cellulose degradation machinery is localised on the cell surface in a substrate dependent fashion [8]. However, this conclusion was based on the identification of a small subset of proteins when compared to the number of proteins annotated in the genome as being involved in cellulose degradation. Therefore, in this study, we addressed the role of proteins in adhesion to and subsequent degradation of cellulose.

We reasoned that localisation of proteins involved in cellulose degradation on the cell envelope, in response to cellulose, should bring out a change in the physicochemical properties of the bacterial cell surface of the cellulose grown cells compared to the glucose grown cells. These changes could be resolved using surface characterisation techniques such as EPM, MATH assay and FTIR [9]. In line with our reasoning, the MATH assay results revealed that the surface of the cellulose grown cells are more hydrophilic compared to the glucose grown cells. This may be due to the expression of cellulose degradation components (GHs, CBPs and non-cellulolytic adherence proteins) on the cell surface of cellulose grown cells. Although current literature suggests that CBPs are hydrophobic, the decrease in hydrophobicity of the cell surface may not be surprising, since the hydrophobic domains of the CBPs may be embedded within the outer membrane with the hydrophilic domains exposed on the surface. Moreover, the MATH assay measures the net surface characteristics of the cell and does not take into account cell surface heterogeneity. Furthermore, a similar decrease in cell surface hydrophobicity upon exposure to cellulose has been previously observed in *Ruminococcus albus* (a cellulosome producing bacterium) [46]. It has been suggested that the decrease in hydrophobicity in *R*. *albus* can be attributed to the production of a pili-protein, which can mediate cellulose adhesion [47, 48].

The role of envelope proteins in cellulose degradation in *F*. *succinogenes* is also supported by the electrophoretic mobility measurements. An increase in the isoelectric point of the cell surface of *F*. *succinogenes* when grown in cellulose may be a consequence of the decrease in cell surface polysaccharides and an increase in the cell surface protein content, as evidenced by the FTIR measurements. Therefore, the results of the colloidal surface characterisation study strongly suggests that cells exposed to cellulose change their surface characteristics by increasing the protein content on their cell surface.

In order to gain a better understanding of the role of proteins in adhesion and cellulose degradation, a proteomic analysis of the surface associated proteins was carried out. A total of 185 cell envelope-associated proteins were identified during growth on both glucose and cellulose, 165 of which are uniquely reported in our study [7, 8, 41–44].

Of the 185 proteins we identified, 25 are predicted to be associated with cellulose degradation. Among these, 5 proteins were identified irrespective of whether the cells were grown in glucose or two different forms of cellulose. The probable changes in the relative expression levels of these proteins across the different substrates can be inferred from the number of peptides identified for each protein in a particular substrate. Using this approach, the data suggest that the localisation of the fibro-slime domain protein (Fisuc_1979) (formerly cellulose binding protein (CBP) of 180-kDa) on the cell surface was increased upon exposure to cellulose; which is in agreement with previous observations [8]. Therefore, the fibro-slime domain protein can be considered as a major non-catalytic CBM protein that facilitates close contact of GHs and CBMs to cellulose substrates during cellulose degradation. This could also suggest that *F*. *succinogenes* might have a different adhesion pattern when compared to *Cytophaga hutchinsonii*, a cellulose degrading bacterium, whose cellulose degradation mechanism *F*. *succinogenes* was thought to follow [49]. Similarly, an increased abundance of a GH family 8 protein (Fisuc_1802) on the cell surface is observed in the presence of cellulose as compared to glucose. This protein may be involved in the hydrolysis of the glycosidic bonds in cellulose, particularly in the crystalline form. Remarkably, the localisation of the extracellular solute-binding protein family 5 (Fisuc_1230) to the cell surface seems to increase only in the presence of MC cellulose and we speculate that this protein may be involved in structural modification of MC cellulose to aid degradation, or may be involved in import of hydrolytic products such as cellodextrins into the cell. A carbohydrate binding family 11 (Fisuc_3111) protein did not seem to significantly vary with change in substrates in this study. Among the 25 proteins identified as playing a possible role in cellulose degradation, 16 proteins are unique to cellulose treatments and we speculate that these proteins are localised on the cell surface in response to cellulose and can play a major role in adhesion and cellulose degradation. Among these, 5 proteins–three cellulases (Fisuc_1224; Fisuc_1523 and Fisuc_2772), one endoglucanase (Fisuc_2361) and one glycoside hydrolase family protein (Fisuc_0393) were found to belong to either GH5 or GH9 family of proteins. The GH5 family of proteins possess conserved glutamic acid residues that are potentially involved in the catalytic mechanism [48], which provides thermal stability to these proteins. Indeed, a previous study on rumen cellulose degraders, similar to *F*. *succinogenes*, demonstrated that GH5 and GH9 enzymes are more versatile and have a remarkable capability to degrade (MC) cellulose [50]. Therefore, the GH5 family of cellulases we identified in *F*. *succinogenes* may have potential advantages for future biofuels generation [51]. The combination of these enzymes perhaps display hydrolytic synergy at the surface and help provide efficient cellulose degradation.

Further evidence that suggests that cellulose degradation occurs at the cell surface is provided by the identification of extracellular solute binding proteins—known to be involved in active transport of solutes across the cytoplasmic membrane—and proteins that possess CBM1, CBM11 and CBM30, which are hypothesised to bind single chains of cellulose [7] localised on the surface of *F*. *succinogenes*. We also identified alpha amylase (Fisuc_1932) only in AS cellulose treatment, which may suggest partial conversion of cellulose to starch during the preparation of AS cellulose [52]. The 43 cell envelope proteins identified as having a role in transport of solute across the membranes include seven OmpA family proteins, five TPR domain proteins, three ABC transporters and three MotA/TolQ/ExbB proteins. OmpA/MotB domain protein (Fisuc_1592) is present as one of the most abundant protein on the cell envelope of *F*. *succinogenes*. A large number of TPR domain proteins, which play an important role in protein-protein interaction and multi-protein complex formation [53], were found on the cell surface of *F*. *succinogenes*. Specifically, the expression of the TPR domain protein (Fisuc_2398) seems to increase in the presence of cellulose and may play an important role in co-ordinating the cellulose degradation process. Identification of a wide variety of transporters suggests that once degradation of cellulose occurs on the cell surface, the degradation products are transported to the periplasm for further metabolism.

In line with this hypothesis, cellodextrin phosphorylase (Fisuc_2900), belonging to GH94 family, was found exclusively in the cells grown on cellulose (and was not detected in glucose grown cells). In anaerobes, like *F*. *succinogenes*, cellodextrin phosphorylase catalyses the ATP independent phosphorolysis reaction and the microorganism can gain energy from phosphorolytic cleavage of -glycosidic bonds when cutting the cellodextrin chain [3, 54] to produce glucose [55]. This protein is able to synthesize and degrade cellodextrins reversibly [56]. Given that cellodextrin degradation is mediated by cellodextrin phosphorylase and that this protein is predicted to be localised within the inner membrane of the cellulose grown cells, it is very likely that once cellulose degradation occurs on the cell surface, cellodextrins–the degradation products of cellulose–enter the periplasm and are further processed to glucose by the cellodextrin phosphorylase.

Further, 117 proteins were identified with other activities, including 68 proteins with unknown functions (S2 Table). The number of cell envelope proteins identified is comparatively higher in cellulose grown cells than in the glucose (control) treatment. Moreover, there are several putative/ uncharacterised proteins (such as Fisuc_2732, FSU_2695, Fisuc_0866, Fisuc_0328, Fisuc_0888, Fisuc_0382, Fisuc_0081, Fisuc_0220, Fisuc_2572, Fisuc_2068, Fisuc_0062, Fisuc_2811, Fisuc_2965, Fisuc_2494) which were only identified or highly abundant in cellulose treatment conditions, which leads us to conclude that these hypothetical proteins may be new families of proteins involved in cellulose adhesion and degradation. To date, the prediction of the function of these cell envelope-associated proteins remains a major research challenge.

In conclusion, this study demonstrates through physicochemical characterisation and proteomics analysis, that the presence of cellulose alters the cell surface protein display of *F*. *succinogenes*. Adhesion to cellulose is mediated by the increased cell surface protein display and a concomitant reduction in the polysaccharide display on the cell surface. The results of this study further indicate that the cellulose degradation machinery in *F*. *succinogenes* may indeed be localised on the cell surface, with active transport of degradation products–cellodextrins—across the outer membrane. Subsequent degradation of cellodextrins to glucose is mediated by cellodextrin phosphorylase, localised on inner cytoplasmic membrane of *F*. *succinogenes*. Although various proteins have been identified in this study, it is still not clear how they work collectively and function in cellulose degradation. Further research is needed at the functional and proteomic / systems biology levels to determine the detailed mechanism of cellulose degradation by this unusual microbe.

## Supporting Information

S1 Fig Transmission electron microscopy (TEM) images of the bacterium *F*. *succinogenes* S85. A and B—cells grown on glucose; C and D—cells grown and attached to cellulose particles.
(TIFF)

S2 Fig Detachment of FS cells from the cellulose particles using 0.1% methyl cellulose treatment. A and B—before treatment and C and D—after treatment.
(TIFF)

S3 Fig SDS PAGE of biotinylated samples from different substrate conditions. Group I; (M) Marker, (AS1-2) AS cellulose, (MC1-2) MC cellulose and (G1-2) Glucose, Group II; (M) Marker, (UB1-2) Unbiotinylated samples.
(TIFF)

S1 Table List of soluble proteins identified in *F*. *succinogenes* S85 with distribution of unique peptides among the treatments. (DOCX)

S2 Table List of membrane associated proteins with unknown functions. (DOCX)

S3 Table Peptide sequences, charge and score. (XLSX)
