# Supplementary figures and images for: Influence of Substrates on the Surface Characteristics and Membrane Proteome of Fibrobacter succinogenes S85
Source: PLoS One. 2015 Oct 22;10(10):e0141197. doi: 10.1371/journal.pone.0141197 (PMC4619616; doi:10.1371/journal.pone.0141197)

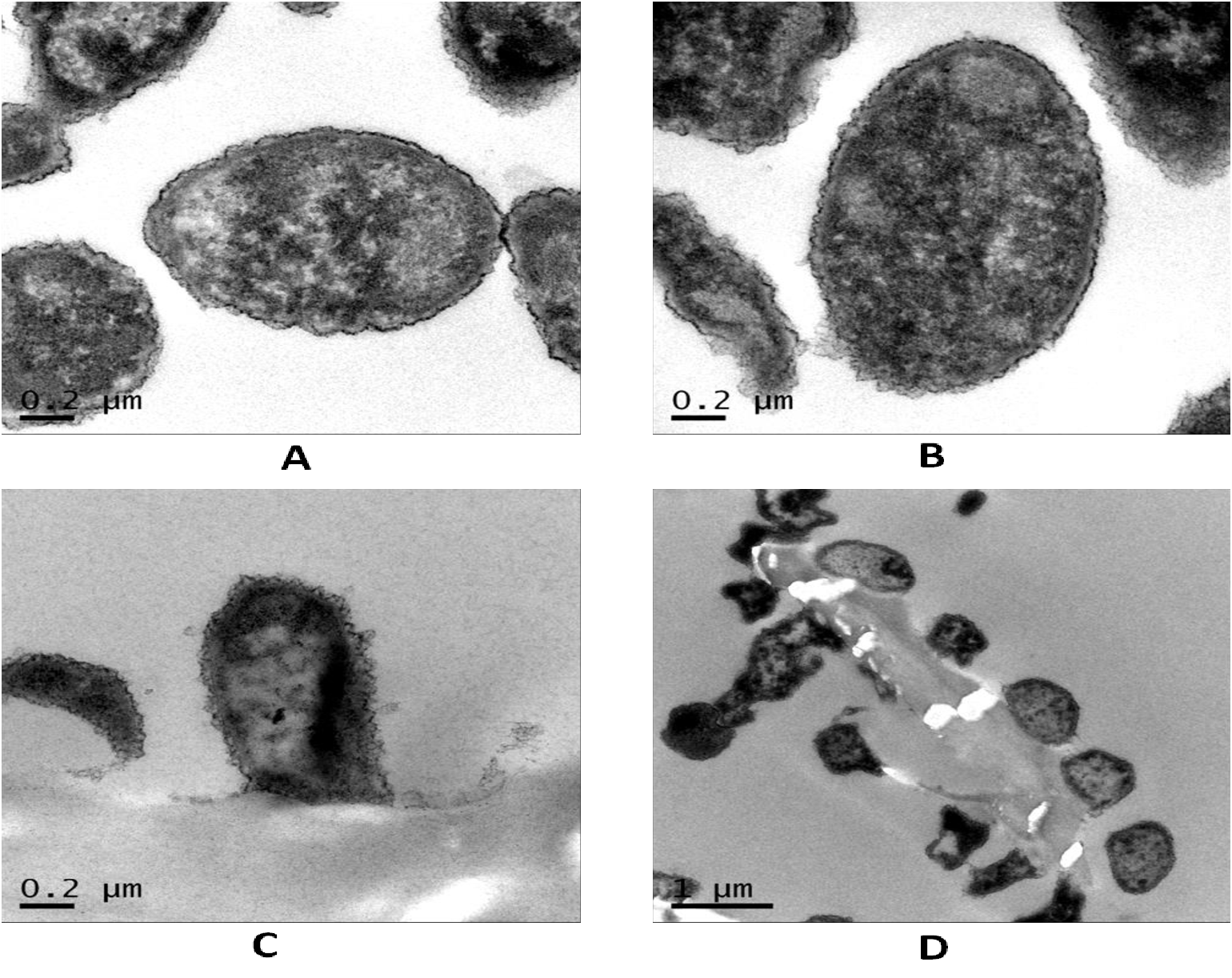

Supplement: S1 Fig — A and B—cells grown on glucose; C and D—cells grown and attached to cellulose particles. (TIFF) [file pone.0141197.s001.tiff]

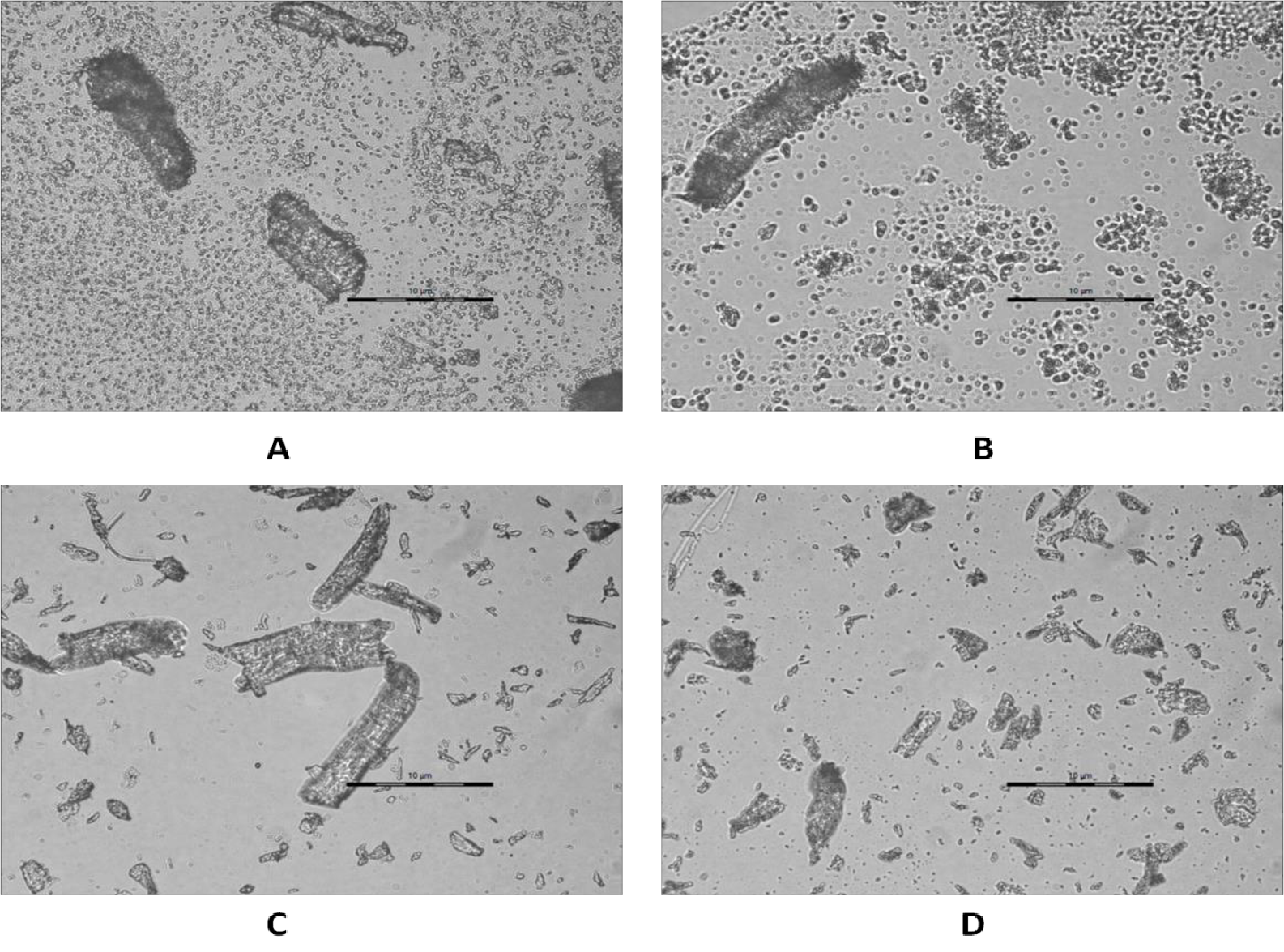

Supplement: S2 Fig — A and B—before treatment and C and D—after treatment. (TIFF) [file pone.0141197.s002.tiff]

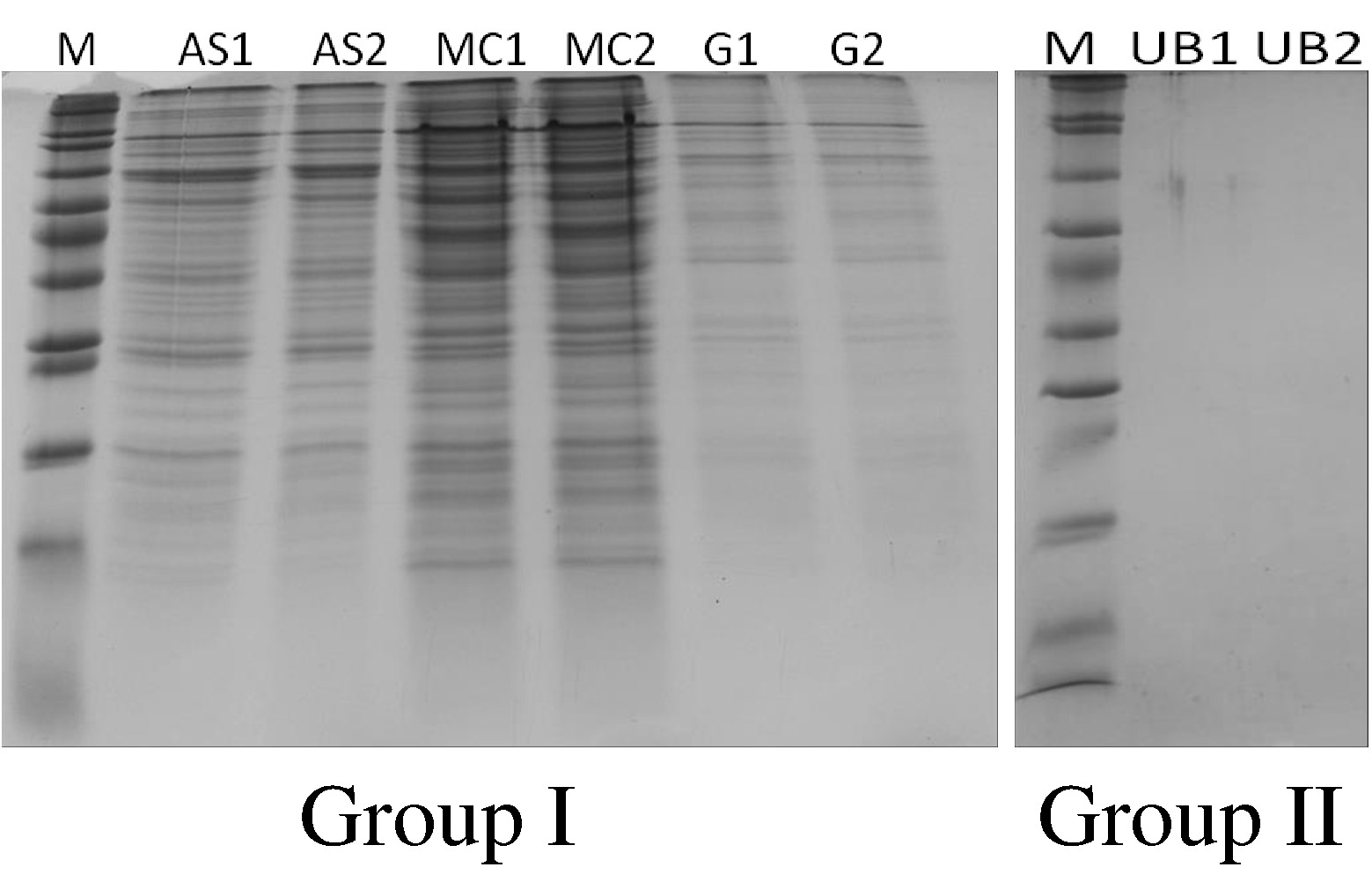

Supplement: S3 Fig — Group I; (M) Marker, (AS1-2) AS cellulose, (MC1-2) MC cellulose and (G1-2) Glucose, Group II; (M) Marker, (UB1-2) Unbiotinylated samples. (TIFF) [file pone.0141197.s003.tiff]
