# Supplementary material for: Influence of Substrates on the Surface Characteristics and Membrane Proteome of Fibrobacter succinogenes S85
Source: PLoS One. 2015 Oct 22;10(10):e0141197. doi: 10.1371/journal.pone.0141197 (PMC4619616; doi:10.1371/journal.pone.0141197)
Supplement: S1 Table — (DOCX) [file pone.0141197.s004.docx]

**Table S1**. List of soluble proteins identified in *F. succinogenes* S85 with distribution of unique peptides among the treatments

| Locus ID | Protein description | Glucose | MC cellulose | AS cellulose |
| --- | --- | --- | --- | --- |
| Fisuc_1922 FSU_2430 | Uncharacterized protein | - | - | 2 |
| Fisuc_1941 FSU_2460 | 50S ribosomal protein L28 | - | - | 3 |
| Fisuc_2030 | Aldo/keto reductase | - | - | 2 |
| Fisuc_2088 | Threonine--tRNA ligase | - | 2 | - |
| Fisuc_2128 FSU_2663 | NADH-quinone oxidoreductase subunit D | - | - | 2 |
| Fisuc_0131 FSU_0534 | RmuC domain protein | - | 2 | - |
| Fisuc_0137 FSU_0540 | Anthranilate synthase | 2 | - | 7 |
| Fisuc_0138 FSU_0541 | Anthranilate phosphoribosyltransferase | - | - | 2 |
| Fisuc_0171 FSU_0574 | Uncharacterized protein | 13 | 19 | 10 |
| Fisuc_0172 | Cysteine desulfurase, SufS subfamily | - | 2 | - |
| Fisuc_0179 FSU_0586 | Tryptophan synthase beta chain | - | - | 3 |
| Fisuc_0181 FSU_0588 | Aconitate hydratase | - | - | 2 |
| Fisuc_0193 FSU_0600 | UDP-N-acetylglucosamine 1-carboxyvinyltransferase | - | - | 2 |
| Fisuc_0205 FSU_0612 | 30S ribosomal protein S4 | 4 | 3 | 7 |
| Fisuc_0211 FSU_0618 | Phosphoribosylaminoimidazole-succinocarboxamide synthase | - | - | 3 |
| Fisuc_2205 | Acetylornithine aminotransferase | - | - | 3 |
| Fisuc_2220 FSU_2762 | Mammalian cell entry related domain protein | - | 2 | - |
| Fisuc_0255 FSU_0665 | Aspartate carbamoyltransferase | - | 2 | 2 |
| Fisuc_0265 FSU_0677 | Chaperone protein HtpG | - | - | 3 |
| Fisuc_0269 FSU_0681 | Pyridoxal biosynthesis lyase | - | 2 | 3 |
| Fisuc_0282 FSU_0693 | Phenylalanine--tRNA ligase beta subunit | 2 | 2 | 3 |
| Fisuc_0313 FSU_0727 | Ketol-acid reductoisomerase | 2 | - | 6 |
| Fisuc_0337 FSU_0752 | Putative thiol methyltransferase | - | - | 2 |
| Fisuc_0338 FSU_0753 | SEC-C domain protein | - | - | 2 |
| Fisuc_0339 FSU_0754 | N-acetyl-gamma-glutamyl-phosphate reductase | - | - | 2 |
| Fisuc_0356 FSU_0771 | Probable transcriptional regulatory protein | - | - | 2 |
| Fisuc_0360 FSU_0775 | 4-hydroxy-tetrahydrodipicolinate synthase | - | - | 4 |
| Fisuc_2295 FSU_2842 | N utilization substance protein A | - | - | 5 |
| Fisuc_2296 FSU_2843 | Translation initiation factor IF-2 | - | - | 3 |
| Fisuc_2368 FSU_2922 | Triosephosphate isomerase (TIM) | 7 | 5 | 20 |
| Fisuc_2388 FSU_2945 | Polyribonucleotide nucleotidyltransferase | 6 | 7 | 11 |
| Fisuc_2392 FSU_2951 | D-isomer specific 2-hydroxyacid dehydrogenase NAD-binding protein | 4 | 7 | 9 |
| Fisuc_2395 FSU_2954 | Serine-tRNA ligase | - | - | 3 |
| Fisuc_2403 FSU_2962 | 50S ribosomal protein L19 | 2 | - | - |
| Fisuc_0381 FSU_0796 | Phosphoglycerate kinase | 6 | 3 | 20 |
| Fisuc_0400 FSU_0815 | Transketolase | - | - | 5 |
| Fisuc_0414 FSU_0828 | Conserved domain protein | - | 2 | - |
| Fisuc_0470 FSU_0888 | Methionine aminopeptidase | - | - | - |
| Fisuc_0475 FSU_0893 | Elongation factor G (EF-G) | 3 | - | - |
| Fisuc_0484 FSU_0902 | 50S ribosomal protein L9 | 2 | 5 | 10 |
| Fisuc_0485 FSU_0903 | 30S ribosomal protein S18 | - | - | 3 |
| Fisuc_0492 FSU_0910 | RNA methyltransferase, TrmH family, group 3 | - | 2 | 4 |
| Fisuc_2406 FSU_2965 | 30S ribosomal protein S16 | 5 | 4 | 8 |
| Fisuc_2439 FSU_3003 | 50S ribosomal protein L25 | 11 | 15 | 14 |
| Fisuc_2482 FSU_3049 | Serine hydroxymethyltransferase (SHMT) | 2 | 7 | 7 |
| Fisuc_2527 | Uncharacterized protein | - | 3 | - |
| Fisuc_2533 FSU_3102 | Elongation factor P (EF-P) | - | - | 2 |
| Fisuc_0579 | Uncharacterized protein | - | - | 2 |
| Fisuc_0581 FSU_1007 | Phosphoserine aminotransferase | - | - | 2 |
| Fisuc_0593 FSU_1020 | Ribosome-binding ATPase YchF | - | - | 2 |
| Fisuc_0620 FSU_1051 | Ornithine carbamoyltransferase | - | - | 2 |
| Fisuc_0636 FSU_1067 | Argininosuccinate synthase | 4 | 7 | 10 |
| Fisuc_2615 FSU_3185 | Malate dehydrogenase | 3 | 2 | 7 |
| Fisuc_2635 FSU_3204 | Lysine--tRNA ligase | - | - | 2 |
| Fisuc_2640 FSU_3209 | Adenylate kinase (AK) | 3 | 2 | 7 |
| Fisuc_2644 FSU_3213 | 3-isopropylmalate dehydrogenase | - | - | 4 |
| Fisuc_2670 FSU_3239 | Conserved hypothetical peptidase | 3 | - | 2 |
| Fisuc_0658 FSU_1089 | Uncharacterized protein | - | - | 4 |
| Fisuc_0718 FSU_1155 | Uncharacterized protein | - | 3 | 5 |
| Fisuc_0721 FSU_1158 | Threonine synthase | - | - | 3 |
| Fisuc_0749 | 30S ribosomal protein S1 | 8 | 9 | 22 |
| Fisuc_0768 FSU_1209 | 2,3-bisphosphoglycerate-independent phosphoglycerate mutase | - | - | 3 |
| Fisuc_2786 FSU_0038 | S-adenosylmethionine synthase (AdoMet synthase) |  | 4 | - |
| Fisuc_2803 | Carboxyl transferase | 4 | 10 | 12 |
| Fisuc_2804 FSU_0059 | Malonyl CoA-acyl carrier protein transacylase | - | - | 5 |
| Fisuc_0838 FSU_1282 | DNA-binding protein HU | - | 3 | 3 |
| Fisuc_0845 FSU_1289 | Putative pyruvate carboxylase, B subunit | 9 | 6 | 5 |
| Fisuc_0889 | Uncharacterized protein | 2 | 9 | 5 |
| Fisuc_0898 | Glutamate synthase (NADPH), homotetrameric | - | - | 6 |
| Fisuc_0899 | Oxidoreductase FAD/NAD(P)-binding domain protein | - | - | 3 |
| Fisuc_2816 FSU_0071 | Conserved domain protein (Uncharacterized protein) | - | - | 3 |
| Fisuc_2835 FSU_0091 | Uncharacterized protein (V-type sodium ATPase, E subunit) | - | 6 |  |
| Fisuc_2837 FSU_0093 | V-type ATP synthase alpha chain | - | 10 | 3 |
| Fisuc_2838 FSU_0094 | V-type ATP synthase beta chain | - | 12 | - |
| Fisuc_2848 | Methionine--tRNA ligase | - | - | 2 |
| Fisuc_2859 FSU_0117 | Transcription-repair-coupling factor (TRCF) | - | - | 3 |
| Fisuc_2866 FSU_0124 | Aspartyl/glutamyl-tRNA(Asn/Gln) amidotransferase subunit B | - | - | 2 |
| Fisuc_2872 FSU_0130 | IMP cyclohydrolase | - | - | 3 |
| Fisuc_2891 FSU_0150 | Fructose-1,6-bisphosphate aldolase, class II | 3 | 3 | 10 |
| Fisuc_2910 | Thiazole synthase (EC 2.8.1.10) | - | - | 3 |
| Fisuc_2921 FSU_0184 | Chaperone protein DnaJ | - | 3 | - |
| Fisuc_2922 FSU_0185 | Chaperone protein DnaK (HSP70) | 6 | - | 7 |
| Fisuc_0947 FSU_1393 | Elongation factor Ts (EF-Ts) | 5 | - | 7 |
| Fisuc_0948 | 30S ribosomal protein S2 | 8 | 5 | 10 |
| Fisuc_0949 FSU_1395 | Electron transfer flavoprotein alpha/beta-subunit | 6 | - | 4 |
| Fisuc_0950 FSU_1396 | Electron transfer flavoprotein alpha subunit | - | - | 2 |
| Fisuc_1004 | DNA-directed RNA polymerase subunit beta | - | 6 | 6 |
| Fisuc_1014 FSU_1461 | Aminodeoxychorismate lyase (Uncharacterized protein) | - | 3 | - |
| Fisuc_1044 FSU_1495 | Formate C-acetyltransferase | - | 2 | 2 |
| Fisuc_2949 FSU_0213 | Phosphoenolpyruvate carboxykinase | 19 | 9 | 24 |
| Fisuc_2964 FSU_0229 | Aspartate-semialdehyde dehydrogenase | - | - | 4 |
| Fisuc_2971 FSU_0236 | Diphosphate--fructose-6-phosphate 1-phosphotransferase | - | 2 | 8 |
| Fisuc_2986 FSU_0251 | Dihydrodipicolinate reductase | - | - | 3 |
| Fisuc_3045 FSU_0310 | Pyridoxal-phosphate dependent TrpB family enzyme | - | - | 2 |
| Fisuc_3047 FSU_0312 | 50S ribosomal protein L21 | 2 | - | 2 |
| Fisuc_3062 FSU_0328 | Glycine-tRNA ligase | 2 | 2 | 4 |
| Fisuc_3075 FSU_0341 | Uncharacterized protein | - | - | 2 |
| Fisuc_1071 FSU_1526 | O-acetylhomoserine sulfhydrylase | - | 5 | 10 |
| Fisuc_1072 FSU_1527 | Cysteine synthase | - |  | 6 |
| Fisuc_1144 FSU_1602 | ATP phosphoribosyltransferase | - | 4 | 4 |
| Fisuc_3113 FSU_0385 | Homoserine O-acetyltransferase | - | 6 |  |
| Fisuc_1204 FSU_1665 | Glucose-6-phosphate isomerase (GPI) | - |  | 2 |
| Fisuc_1209 FSU_1670 | ATPase associated with various cellular activities AAA_3 | - |  | 2 |
| Fisuc_1236 | Mammalian cell entry related domain protein | - | 2 |  |
| Fisuc_1261 FSU_1724 | Enolase | 3 | 16 | 14 |
| Fisuc_1268 FSU_1731 | 30S ribosomal protein S7 | 5 | 5 | 5 |
| Fisuc_1269 | 30S ribosomal protein S12 | 3 | 2 | 3 |
| Fisuc_1270 FSU_1733 | DNA-directed RNA polymerase subunit beta' (RNAP subunit beta') | 10 | 10 | 13 |
| Fisuc_1271 FSU_1734 | DNA-directed RNA polymerase subunit beta (RNAP subunit beta) | 6 | 13 | 8 |
| Fisuc_1273 FSU_1736 | 50S ribosomal protein L10 | 2 | - | 4 |
| Fisuc_1274 FSU_1737 | 50S ribosomal protein L1 | 4 | 7 | 6 |
| Fisuc_1275 FSU_1738 | 50S ribosomal protein L11 | 2 | 2 | 5 |
| Fisuc_1283 FSU_1751 | Uncharacterized protein | - | 2 | 5 |
| Fisuc_1289 FSU_1757 | Putative type III restriction-modification system, Res subunit | 2 | - | - |
| Fisuc_1320 FSU_1787 | DNA-binding response regulator | - | - | 4 |
| Fisuc_1325 FSU_1793 | Phosphate acetyltransferase | - | - | 4 |
| Fisuc_1374 FSU_1840 | Aspartokinase | 2 | - | - |
| Fisuc_1388 FSU_1855 | ATP-dependent Clp protease ATP-binding subunit ClpX | - | - | 2 |
| Fisuc_1389 FSU_1856 | ATP-dependent Clp protease proteolytic subunit | - | - | 2 |
| Fisuc_1390 FSU_1857 | Trigger factor (TF) | 6 | - | 9 |
| Fisuc_1396 | 50S ribosomal protein L17 | 4 | 8 | 8 |
| Fisuc_1397 | DNA-directed RNA polymerase subunit alpha (RNAP subunit alpha) | - | 5 | 3 |
| Fisuc_1398 FSU_1866 | 30S ribosomal protein S11 | 3 | 6 | 6 |
| Fisuc_1399 FSU_1867 | 30S ribosomal protein S13 | - | - | 2 |
| Fisuc_1403 FSU_1871 | 50S ribosomal protein L15 | 4 | 3 | 7 |
| Fisuc_1405 | 30S ribosomal protein S5 | 7 | 5 | 7 |
| Fisuc_1406 FSU_1874 | 50S ribosomal protein L18 | - | 2 | - |
| Fisuc_1407 FSU_1875 | 50S ribosomal protein L6 | 7 | 10 | 14 |
| Fisuc_1408 FSU_1876 | 30S ribosomal protein S8 | - | - | 2 |
| Fisuc_1410 FSU_1878 | 50S ribosomal protein L5 | 9 | 7 | 7 |
| Fisuc_1412 | 50S ribosomal protein L14 | 4 | 2 | 5 |
| Fisuc_1415 FSU_1883 | 50S ribosomal protein L16 | 2 | - | 3 |
| Fisuc_1416 FSU_1884 | 30S ribosomal protein S3 | 9 | 2 | 4 |
| Fisuc_1417 FSU_1885 | 50S ribosomal protein L22 | - | 2 | 3 |
| Fisuc_1419 FSU_1887 | 50S ribosomal protein L2 | 6 | 3 | 8 |
| Fisuc_1420 | 50S ribosomal protein L23 | - | 2 | - |
| Fisuc_1421 FSU_1889 | 50S ribosomal protein L4 | 7 | 8 | 11 |
| Fisuc_1422 FSU_1890 | 50S ribosomal protein L3 | 4 | 3 | 8 |
| Fisuc_1423 FSU_1891 | 30S ribosomal protein S10 | - | - | 2 |
| Fisuc_1461 FSU_1934 | Protein translocase subunit SecA | - | 3 | 3 |
| Fisuc_1472 FSU_1946 | Phosphoribosylformylglycinamidine cyclo-ligase | 2 | - | 2 |
| Fisuc_1486 FSU_1967 | Rubredoxin domain/rubrerythrin domain protein (Rubrerythrin) | - | - | 2 |
| Fisuc_1572 FSU_2059 | PTS system, IIA component | - | - | 2 |
| Fisuc_1627 FSU_2115 | Adenylosuccinate synthetase (AMPSase) | 2 | 2 | 4 |
| Fisuc_1629 | PASTA domain containing protein | 2 | - | 5 |
| Fisuc_1646 FSU_2135 | Geranylgeranyl diphosphate synthase | - | - | 2 |
| Fisuc_1648 FSU_2137 | RNA methyltransferase, TrmH family | - | - | 3 |
| Fisuc_1805 FSU_2306 | Isoleucine--tRNA ligase | - | - | 2 |
| FSU_2617 | Threonine--tRNA ligase | - | - | 7 |
| FSU_2666 | NADH-quinone oxidoreductase, G subunit (EC 1.6.99.5) | 2 | - | 3 |
| FSU_0436 | Uncharacterized protein | 2 | - | 5 |
| FSU_0664 | Putative dihydroorotase, multifunctional complex type | - | 2 | 2 |
| FSU_0773 | Phosphoglucomutase/phosphomannomutase family protein | - | - | 2 |
| FSU_1005 | Conserved domain protein | - | 3 | - |
| FSU_1080 | Branched-chain-amino-acid transaminase | - | - | 6 |
| FSU_1109 | Uncharacterized protein | - | 5 | - |
| FSU_2136 | 1-deoxy-D-xylulose-5-phosphate synthase | - | 2 | - |
| Fisuc_1279 FSU_1743 | Elongation factor Tu (EF-Tu) | 11 | 10 | 20 |

**Table S2**. List of membrane associated proteins with unknown functions

| Locus ID | Protein description | Glucose | MC Cellulose | AS cellulose | Location^a^ | Gravy index^b^ | Molecular mass (kDa)^b^ | pI^b^ | Presence of signal peptide (amino acid position)^C^ | Reference |
| --- | --- | --- | --- | --- | --- | --- | --- | --- | --- | --- |
| Fisuc_2370 FSU_2924 | Putative lipoprotein | - | - | 3 | Unknown | -0.59 | 16.756 | 5.37 | No | - |
| Fisuc_0043 | Putative uncharacterized protein | - | 7 |  | Non cytoplasmic | -0.278 | 29.93 | 9.3 | Yes (24-25) | - |
| Fisuc_0067 FSU_0466 | 3-isopropylmalate dehydratase large subunit | - | - | 3 | Unknown | -0.232 | 50.913 | 6.07 | No | - |
| Fisuc_0068 FSU_0467 | 3-isopropylmalate dehydratase, small subunit | - | - | 3 | Unknown | -0.163 | 22.009 | 6.21 | No | - |
| Fisuc_2016 FSU_2539 | 3-oxoacyl-(Acyl-carrier-protein) reductase | 4 | - | - | Unknown | 0.094 | 25.131 | 6.35 | No | [8] |
| Fisuc_3070 FSU_0336 | 3-oxoacyl-[acyl-carrier-protein] synthase 2 | - | - | 2 | Cytoplasmic membrane | -0.049 | 43.761 | 5.67 | No | - |
| Fisuc_2494 FSU_3062 | 4Fe-4S ferredoxin iron-sulfur binding domain protein | - | 13 | 8 | Cytoplasmic membrane | -0.102 | 28 | 6.52 | No | - |
| Fisuc_2502 FSU_3070 | 4Fe-4S ferredoxin iron-sulfur binding domain protein | 13 |  | - | Cytoplasmic membrane | -0.115 | 27.52 | 6.52 | No | - |
| Fisuc_0975 FSU_1421 | 50S ribosomal protein L13 | 3 | 4 | 5 | Periplasm | -0.113 | 15.6 | 9.8 | No | - |
| FSU_0628 | 50S ribosomal protein L31 | - | - | 2 | Unknown | -0.827 | 9.949 | 9.47 | No | - |
| Fisuc_1272 | 50S ribosomal protein L7/L12 | 2 | 2 | 5 | Non cytoplasmic | 0.206 | 12.792 | 5.27 | No | - |
| Fisuc_0061 FSU_0456 | 60 kDachaperonin | 5 | 7 | 5 | Unknown | -0.128 | 57.52 | 5.41 | No | - |
| Fisuc_2432 FSU_2995 | Adenylate/guanylate cyclase domain protein | - | - | 2 | Cytoplasmic membrane | -0.341 | 115.294 | 6.03 | No | - |
| Fisuc_0655 | Aminotransferase class I and II | - | - | 2 | Unknown | -0.102 | 47.04 | 6.1 | No | - |
| Fisuc_2778 | ATP-dependent zinc metalloproteaseFtsH 1 | - | 3 | - | Cytoplasmic membrane | -0.37 | 77.197 | 6.04 | No | - |
| Fisuc_1010 FSU_1457 | BatA protein | - | 2 | - | Cytoplasmic membrane | 0.013 | 40.98 | 9.18 | No | - |
| Fisuc_1008 FSU_1454 | BatB protein | - | 2 | - | Cytoplasmic membrane | -0.006 | 38.346 | 9.54 | No | - |
| Fisuc_0851 FSU_1295 | Carboxyl-terminal protease | - | - | 3 | Cytoplasmic membrane | -0.359 | 66.051 | 9.19 | Yes (24-25) | - |
| Fisuc_2588 FSU_3158 | Conserved domain protein | - | 5 | 2 | Cytoplasmic membrane | -0.201 | 64.29 | 8.22 | No | - |
| Fisuc_0841 FSU_1285 | Conserved domain protein | - | - | 2 | Unknown | -0.197 | 27.296 | 6.91 | No | - |
| Fisuc_0002 FSU_0395 | Diaminopimelate dehydrogenase | - | - | 4 | Unknown | -0.17 | 35.752 | 6.76 | No | - |
| Fisuc_0777 FSU_1218 | dTDP-4-dehydrorhamnose 3,5-epimerase | - | - | 2 | Unknown | -0.327 | 21.178 | 5.33 | No | - |
| FSU_2120 | FG-GAP repeat protein | - | - | 2 | Unknown | -0.216 | 121.23 | 5.79 | No | - |
| Fisuc_1632 | FG-GAP repeat protein | - | 2 |  | Non cytoplasmic | -0.193 | 122.994 | 5.93 | Yes (23-24) | - |
| Fisuc_2905 FSU_0167 | FKBP-type peptidyl-prolylcis-trans isomerase domain protein | 4 | 6 | 5 | Periplasm | -0.496 | 48.48 | 8.83 | Yes (21-22) | - |
| Fisuc_2811 FSU_0066 | Glu/Leu/Phe/Val dehydrogenase | - | 5 | 10 | Unknown (multiple location) | -0.235 | 48.622 | 6.86 | No | - |
| Fisuc_0016 FSU_0409 | GTP-binding protein TypA | - | - | 3 | Cytoplasmic membrane | -0.291 | 67.979 | 5.16 | No | - |
| Fisuc_2253 FSU_2798 | Homoserine dehydrogenase | - | - | 5 | Unknown | 0.142 | 45.884 | 5.9 | No | - |
| Fisuc_1750 FSU_2248 | Inner membrane protein oxaA | 3 |  | - | Cytoplasmic membrane | 0.023 | 67.957 | 9.09 | No | - |
| Fisuc_1429 FSU_1897 | LemA family protein | - | 3 | - | Unknown | 0.068 | 20.494 | 7.81 | No | - |
| Fisuc_1456 FSU_1929 | LemA family protein | 2 | 2 | - | Unknown | -0.109 | 22.275 | 8.77 | No | - |
| Fisuc_0202 FSU_0609 | Lipoprotein | 3 |  | - | Non cytoplasmic | -0.501 | 19.76 | 5.01 | Yes (19-20) | [8] |
| FSU_2141 | Mce-like protein | - | 2 | - | Unknown | -0.013 | 35.369 | 5.23 | No | - |
| Fisuc_2965 FSU_0230 | Membrane protein | 17 | 5 | 5 | Non cytoplasmic | -0.207 | 44.92 | 4.64 | Yes (19-20) | - |
| Fisuc_1898 FSU_2404 | Membrane protein | 5 | 8 | 5 | Non cytoplasmic | -0.268 | 48.26 | 4.79 | No | [8] |
| Fisuc_1527 FSU_2009 | Membrane protein | 10 | 7 | 7 | Outer membrane | -0.263 | 78.75 | 5.09 | Yes (25-26) | [8] |
| Fisuc_1528 FSU_2010 | Membrane protein | 6 | 4 | 3 | Non cytoplasmic | -0.195 | 78.195 | 5.64 | Yes ( | [8] |
| Fisuc_0242 FSU_0652 | MORN variant repeat protein | - | - | 2 | Non cytoplasmic | -0.658 | 34.161 | 6.96 | Yes (17-18) | - |
| Fisuc_0392 | Mucin-associated surface protein (MASP) | 2 | - | 3 | Cytoplasmic membrane | 0.085 | 22.813 | 4.75 | Yes (17-18) | - |
| Fisuc_2130 FSU_2665 | NADH dehydrogenase (Quinone) | - | 2 | - | Unknown | -0.065 | 46.835 | 6.02 | No | - |
| Fisuc_2129 FSU_2664 | NADH dehydrogenase (Ubiquinone) 24 kDa subunit | - | 2 |  | Unknown | -0.212 | 37.941 | 6.75 | No | - |
| Fisuc_2126 FSU_2661 | NADH-quinoneoxidoreductase subunit B | - | 2 |  | Cytoplasmic membrane | -283 | 23.97 | 6.62 | No | - |
| Fisuc_2059 FSU_2587 | Oxidoreductase domain protein | - | - | 2 | Periplasm | -0.306 | 45.143 | 8.41 | No | - |
| Fisuc_0062 FSU_0457 | Penicillin-binding protein 1A | - | 5 | 4 | Unknown | -0.394 | 90.22 | 9.33 | No | - |
| Fisuc_1443 FSU_1914 | Peptidase M23 | - |  | 2 | Outer membrane | -0.327 | 30.171 | 9.65 | No | - |
| Fisuc_0871 FSU_1317 | Peptidase M23 | - | 2 | 2 | Non cytoplasmic | -0.201 | 48.044 | 9.71 | No | - |
| Fisuc_0872 FSU_1318 | Peptidyl-prolylcis-trans isomerase | 7 | 8 | 7 | Outer membrane | -0.451 | 29.67 | 7.65 | No | - |
| Fisuc_1756 FSU_2256 | Peptidyl-prolylcis-trans isomerase, FKBP-type | 2 | - | - | Periplasm | -0.115 | 42.84 | 6.36 | Yes (18-19) | - |
| Fisuc_0518 FSU_0941 | Peptidyl-prolylcis-trans isomeraseSurA | 3 | - | - | Periplasm | -0.31 | 48.24 | 6.63 | Yes (19-20) | - |
| Fisuc_0775 FSU_1216 | Polysaccharide biosynthesis/export protein | 6 | 8 | 3 | Out ermembrane | -0.181 | 41.49 | 5.74 | Yes (21-22) | - |
| Fisuc_2762 | PpiC-type peptidyl-prolylcis-trans isomerase | 16 | 18 | 11 | Outer membrane | -0.291 | 71.067 | 5.11 | No | - |
| Fisuc_2974 FSU_0239 | PPIC-type PPIASE domain protein | 10 |  | 3 | Non cytoplasmic | -0.422 | 36.986 | 9.5 | Yes (22-23) | - |
| Fisuc_2068 FSU_2596 | Putative lipoprotein | 8 | 20 | 13 | Unknown | -0.539 | 39.21 | 8.81 | No | - |
| Fisuc_2490 FSU_3058 | Putative lipoprotein | - | 7 | 3 | Unknown | -0.551 | 80.441 | 6.59 | Yes (18-19) | - |
| Fisuc_2493 FSU_3061 | Putative lipoprotein | 17 | 33 | 13 | Cytoplasmic membrane | -0.363 | 70.572 | 6.93 | No | - |
| Fisuc_2572 FSU_3142 | Putative lipoprotein | - | 3 | 3 | Periplasm | -0.205 | 71.611 | 5.67 | Yes (21-22) | - |
| Fisuc_0657 FSU_1088 | Putative lipoprotein | - | 2 |  | Non cytoplasmic | -0.103 | 34.721 | 4.56 | Yes (22-23) | - |
| Fisuc_0752 FSU_1190 | Putative lipoprotein | 2 | - | 2 | Unknown | -0.669 | 22.029 | 4.67 | Yes (21-22) | - |
| Fisuc_1141 FSU_1599 | Putative lipoprotein | - | - | 2 | Unknown | -0.391 | 45.816 | 5.28 | Yes (21-22) | - |
| FSU_2769 | putative lipoprotein | - | 2 |  | unknown | -0.408 | 35.975 | 6.52 | Yes (17-18) |  |
| Fisuc_0220 FSU_0627 | Putative lipoprotein | 4 | 10 | 9 | Unknown | -0.373 | 32.7 | 9.54 | No | - |
| Fisuc_0756 FSU_1194 | Putative lipoprotein | - | - | 2 | Periplasm | -0.128 | 32.275 | 8.54 | Yes (22-23) | - |
| Fisuc_0767 FSU_1207 | Putative lipoprotein | 4 | 5 | 5 | Non cytoplasmic | -0.105 | 37.411 | 4.38 | Yes (24-25) | - |
| Fisuc_2795 FSU_0049 | Putative lipoprotein | 2 | 3 | 3 | Non cytpplasmic | -0.79 | 12.637 | 5.48 | Yes (23-24) | - |
| Fisuc_2897 FSU_0158 | Putative lipoprotein | 2 | - | 4 | Non cytoplasmic | -0.017 | 25.293 | 5.3 | Yes (19-20) | - |
| Fisuc_1021 FSU_1468 | Putative lipoprotein | 4 | 3 | 5 | Non cytoplasmic | -0.41 | 19.71 | 6.72 | Yes (19-20) | - |
| Fisuc_3024 FSU_0289 | Putative lipoprotein | - | - | 2 | Unknown | -0.16 | 31.238 | 4.86 | Yes (5-6) | - |
| Fisuc_1317 FSU_1784 | Putative lipoprotein | - | - | 2 | Non cytoplasmic | -0.371 | 22.441 | 5.18 | Yes (20-21) | - |
| Fisuc_1529 FSU_2011 | Putative lipoprotein | 3 | 3 |  | Non cytoplasmic | -0.279 | 24.182 | 4.8 | Yes (23-24) | - |
| FSU_0522 | Putative lipoprotein | - | 2 |  | Non cytoplasmic | -0.408 | 35.975 | 6.52 | Yes (17-18) | - |
| Fisuc_2739 FSU_3310 | Putative membrane protein | 8 | - | 3 | Cytoplasmic membrane | 0.119 | 115.48 | 6.13 | No | - |
| Fisuc_0081 FSU_0479 | Putative uncharacterized protein | - | 5 | 3 | Unknown | -0.151 | 20.617 | 9.01 | No | - |
| Fisuc_2147 | Putative uncharacterized protein | 8 |  | 8 | Unknown | -0.111 | 24.82 | 6.34 | No | - |
| Fisuc_2269 | Putative uncharacterized protein | 3 | 5 | 5 | Unknown | -0.227 | 26.82 | 10.02 | No | - |
| Fisuc_0382 FSU_0797 | Putative uncharacterized protein | - | 2 | 2 | Cytoplasmic membrane | -0.117 | 37.419 | 5.02 | Yes (18-19) | - |
| Fisuc_0482 FSU_0900 | Putative uncharacterized protein | - | - | 2 | Outer membrane | -0.259 | 54.535 | 5.68 | Yes (21-22) | - |
| Fisuc_0820 FSU_1263 | Putative uncharacterized protein | - | - | 2 | Unknown | -0.295 | 26.781 | 5.36 | No | - |
| Fisuc_0888 FSU_1335 | Putative uncharacterized protein | - | 8 | 3 | Non cytoplasmic | -0.487 | 51.75 | 5.28 | Yes (17-18) | - |
| Fisuc_1013 FSU_1460 | Putative uncharacterized protein | - | 3 | - | Non cytoplasmic | -0.303 | 16.368 | 4.8 | Yes (21-22) | - |
| Fisuc_1203 FSU_1664 | Putative uncharacterized protein | - | 8 | 4 | Unknown | -0.34 | 26.03 | 7.75 | No | - |
| Fisuc_1319 FSU_1786 | Putative uncharacterized protein | 2 | - | 4 | Non cytoplasmic | 0.183 | 27.32 | 5.58 | Yes (19-20) | - |
| Fisuc_1526 FSU_2008 | Putative uncharacterized protein | 4 | 3 | 4 | Non cytoplasmic | -0.338 | 85.239 | 5.5 | Yes (19-20) | - |
| Fisuc_1597 | Putative uncharacterized protein | 2 | 6 | 2 | Non cytoplasmic | 0.144 | 29.528 | 9.01 | Yes (22-23) | - |
| FSU_0247 | Putative uncharacterized protein | - | 2 | - | Outer membrane | -0.23 | 34.983 | 4.8 | No | - |
| FSU_3096 | Putative uncharacterized protein | 2 |  | 3 | Unknown | -0.316 | 34.113 | 5.24 | No | - |
| FSU_0881 | Putative uncharacterized protein | 3 | - | - | Non cytoplasmic | -0.585 | 41.95 | 6.35 | Yes (19-20) | - |
| Fisuc_1875 FSU_2377 | Putative uncharacterized protein | - | - | 4 | Non cytoplasmic | -0.5 | 37.593 | 5.53 | Yes (18-19) | - |
| Fisuc_1954 FSU_2474 | Putative uncharacterized protein | - | 4 | - | Extracellular | -0.26 | 64.462 | 6.63 | No | - |
| Fisuc_2020 FSU_2544 | Putative uncharacterized protein | 2 |  | 3 | Non cytoplasmic | -0.055 | 28.17 | 5.1 | Yes (18-19) | - |
| Fisuc_2072 FSU_2600 | Putative uncharacterized protein | 2 | - | 2 | Outer membrane | -0.117 | 38.859 | 6.05 | Yes (23-24) | - |
| Fisuc_0328 FSU_0743 | Putative uncharacterized protein | - | 2 | 2 | Periplasm | -0.314 | 25.13 | 6.63 | No | - |
| Fisuc_2326 | Putative uncharacterized protein | - | - | 3 | Non cytoplasmic | -0.554 | 23.324 | 8.7 | Yes (34-35) | - |
| Fisuc_0474 FSU_0892 | Putative uncharacterized protein | - | 2 | - | Unknown | 0.11 | 24.669 | 5.58 | No | - |
| Fisuc_2506 FSU_3074 | Putative uncharacterized protein | 3 | - | - | Non cytoplasmic | -0.391 | 34.551 | 4.51 | Yes (21-22) | - |
| Fisuc_2544 FSU_3113 | Putative uncharacterized protein | - | 4 | - | Unknown | -0.026 | 36.422 | 9.55 | No | - |
| Fisuc_2555 FSU_3125 | Putative uncharacterized protein | 2 | 2 |  | Non cytoplasmic | -1.191 | 18.92 | 9.06 | Yes (21-22) | - |
| Fisuc_2763 FSU_0015 | Putative uncharacterized protein | 2 | - | - | Non cytoplasmic | -0.339 | 39.8 | 7.62 | Yes (21-22) | - |
| Fisuc_0861 | Putative uncharacterized protein | - | 2 | - | Unknown | -0.354 | 67.25 | 9.38 | Yes (17-18) | - |
| Fisuc_0866 | Putative uncharacterized protein | - | 3 | 3 | Unknown | -0.534 | 264.045 | 5.11 | No | - |
| Fisuc_2863 | Putative uncharacterized protein | 7 | 10 | 10 | Non cytoplasmic | -0.228 | 56.8 | 5.06 | Yes (17-18) | - |
| Fisuc_3015 FSU_0280 | Putative uncharacterized protein | 3 | 2 | 2 | Non cytoplasmic | -0.229 | 37.819 | 5.41 | Yes (20-21) | - |
| Fisuc_3021 | Putative uncharacterized protein | 3 |  |  | Cytoplasmic membrane | -0.005 | 67.29 | 4.51 | No | - |
| Fisuc_1223 FSU_1684 | Putative uncharacterized protein | - | - | 3 | Non cytoplasmic | 0.167 | 31.235 | 5.21 | Yes (19-20) | - |
| Fisuc_1476 | Putative uncharacterized protein | 4 | - | - | Non cytoplasmic | -0.486 | 43.31 | 8.83 | Yes (20-21) | - |
| Fisuc_1485 FSU_1966 | Putative uncharacterized protein | 4 |  |  | Non cytoplasmic | -0.184 | 21.636 | 8.98 | Yes (21-22) | - |
| FSU_2597 | Putative uncharacterized protein | 2 | - | - | Outer membrane | -0.22 | 41.786 | 4.64 | No | - |
| FSU_2684 | Putative uncharacterized protein | - | 8 | - | Cytoplasmic membrane | -0.1 | 26.421 | 6.93 | No | - |
| FSU_2695 | Putative uncharacterized protein | - | 6 | 2 | Unknown | 0.018 | 27.822 | 8.39 | No | - |
| FSU_2876 | Putative uncharacterized protein | - | 2 | - | Non cytoplasmic | -0.559 | 25.727 | 9.18 | No | - |
| FSU_1004 | Putative uncharacterized protein | 2 | - | - | Unknown | -0.618 | 127.845 | 4.62 | No | - |
| Fisuc_2881 FSU_0139 | Pyruvate-flavodoxinoxidoreductase | 7 | 7 | 17 | Unknown | -0.138 | 129.294 | 7.23 | No | - |
| Fisuc_2732 FSU_3303 | Rhodanese domain protein | - | 5 | 4 | Non cytoplasmic | 0.033 | 15.785 | 8.81 | Yes (20-21) | - |
| Fisuc_2492 FSU_3060 | Succinate dehydrogenase | 2 | 3 | 2 | Cytoplasmic membrane | 0.404 | 31.59 | 9.04 | No | - |
| Fisuc_0663 FSU_1094 | Toluene tolerance family protein | 3 | - | - | Non cytoplasmic | -0.449 | 21.87 | 9.22 | Yes (18-19) | - |
| Fisuc_0033 FSU_0426 | Uncharacterized protein | 2 | 4 | 2 | Unknown | -0.711 | 22.89 | 10.11 | No | - |
| Fisuc_2384 FSU_2941 | UPFO365 protein | 2 | 2 |  | Unknown | 0.265 | 34.8 | 7.76 | No | - |
| Fisuc_0556 FSU_0980 | UPFO365 protein | - | - | 2 | Cytoplasmic membrane | -0.082 | 42.916 | 5.68 | No | - |

^a^Location of the given proteins predicted by the PSORTb subcellular localization prediction tool version 3.0 [5]

^b^Theoretical isoelectric point, molecular mass and gravy index of the given protein, as predicted by the ExPASy Compute pI/MW tool [6]

^c^Determined by SignalP v.3.0 [7] the numbers in parentheses indicates the amino acids between which cleavage is predicted to occur in the given protein

1. Miron J, Ben-Ghedalia D, Morrison M (2001) Invited review: adhesion mechanisms of rumen cellulolytic bacteria. J Dairy Sci84: 1294-1309.
2. Jun HS, Qi M, Gong J, Egbosimba EE, Forsberg CW (2007) Outer membrane proteins of *Fibrobacter succinogenes* with potential roles in adhesion to cellulose and in cellulose digestion. J Bacteriol 189: 6806-6815.
3. Kudo H, Cheng KJ, Costerton JW (1987) Electron microscopic study of the methylcellulose-mediated detachment of cellulolytic rumen bacteria from cellulose fibers. Can J Microbiol 33: 267-272.
4. Weimer PJ, Odt CL (1995) Cellulose degradation by ruminal microbes: physiological and hydrolytic diversity among ruminal cellulolytic bacteria. ACS Sym Ser291-304.
5. Nancy YY, Wagner JR, Laird MR, Melli G, Rey S, et al. (2010) PSORTb 3.0: improved protein subcellular localization prediction with refined localization subcategories and predictive capabilities for all prokaryotes. Bioinformatics 26: 1608-1615.
6. Gasteiger E, Hoogland C, Gattiker A, Duvaud S, Wilkins MR, et al. (2005) Protein identification and analysis tools on the ExPASy server. The proteomics protocols handbook: 571-607.
7. Dyrlov Bendtsen J, Nielsen H, von Heijne G, Brunak S (2004) Improved prediction of signal peptides: SignalP 3.0. J Mol Biol 340: 783-795.
