# Supplementary material for: Influence of Substrates on the Surface Characteristics and Membrane Proteome of Fibrobacter succinogenes S85
Source: PLoS One. 2015 Oct 22;10(10):e0141197. doi: 10.1371/journal.pone.0141197 (PMC4619616; doi:10.1371/journal.pone.0141197)
Supplement: S2 Table — (DOCX) [file pone.0141197.s005.docx]

**S2 Table. List of membrane associated proteins with unknown functions**

| Locus ID | Protein description | Glucose | MC Cellulose | AS cellulose | Location^a^ | Gravy index^b^ | Molecular mass (kDa)^b^ | pI^b^ | Presence of signal peptide (amino acid position)^C^ | Reference |
| --- | --- | --- | --- | --- | --- | --- | --- | --- | --- | --- |
| Fisuc_2370 FSU_2924 | Putative lipoprotein | - | - | 3 | Unknown | -0.59 | 16.756 | 5.37 | No | - |
| Fisuc_0043 | Putative uncharacterized protein | - | 7 |  | Non cytoplasmic | -0.278 | 29.93 | 9.3 | Yes (24-25) | - |
| Fisuc_0067 FSU_0466 | 3-isopropylmalate dehydratase large subunit | - | - | 3 | Unknown | -0.232 | 50.913 | 6.07 | No | - |
| Fisuc_0068 FSU_0467 | 3-isopropylmalate dehydratase, small subunit | - | - | 3 | Unknown | -0.163 | 22.009 | 6.21 | No | - |
| Fisuc_2016 FSU_2539 | 3-oxoacyl-(Acyl-carrier-protein) reductase | 4 | - | - | Unknown | 0.094 | 25.131 | 6.35 | No | [8] |
| Fisuc_3070 FSU_0336 | 3-oxoacyl-[acyl-carrier-protein] synthase 2 | - | - | 2 | Cytoplasmic membrane | -0.049 | 43.761 | 5.67 | No | - |
| Fisuc_2494 FSU_3062 | 4Fe-4S ferredoxin iron-sulfur binding domain protein | - | 13 | 8 | Cytoplasmic membrane | -0.102 | 28 | 6.52 | No | - |
| Fisuc_2502 FSU_3070 | 4Fe-4S ferredoxin iron-sulfur binding domain protein | 13 |  | - | Cytoplasmic membrane | -0.115 | 27.52 | 6.52 | No | - |
| Fisuc_0975 FSU_1421 | 50S ribosomal protein L13 | 3 | 4 | 5 | Periplasm | -0.113 | 15.6 | 9.8 | No | - |
| FSU_0628 | 50S ribosomal protein L31 | - | - | 2 | Unknown | -0.827 | 9.949 | 9.47 | No | - |
| Fisuc_1272 | 50S ribosomal protein L7/L12 | 2 | 2 | 5 | Non cytoplasmic | 0.206 | 12.792 | 5.27 | No | - |
| Fisuc_0061 FSU_0456 | 60 kDachaperonin | 5 | 7 | 5 | Unknown | -0.128 | 57.52 | 5.41 | No | - |
| Fisuc_2432 FSU_2995 | Adenylate/guanylate cyclase domain protein | - | - | 2 | Cytoplasmic membrane | -0.341 | 115.294 | 6.03 | No | - |
| Fisuc_0655 | Aminotransferase class I and II | - | - | 2 | Unknown | -0.102 | 47.04 | 6.1 | No | - |
| Fisuc_2778 | ATP-dependent zinc metalloproteaseFtsH 1 | - | 3 | - | Cytoplasmic membrane | -0.37 | 77.197 | 6.04 | No | - |
| Fisuc_1010 FSU_1457 | BatA protein | - | 2 | - | Cytoplasmic membrane | 0.013 | 40.98 | 9.18 | No | - |
| Fisuc_1008 FSU_1454 | BatB protein | - | 2 | - | Cytoplasmic membrane | -0.006 | 38.346 | 9.54 | No | - |
| Fisuc_0851 FSU_1295 | Carboxyl-terminal protease | - | - | 3 | Cytoplasmic membrane | -0.359 | 66.051 | 9.19 | Yes (24-25) | - |
| Fisuc_2588 FSU_3158 | Conserved domain protein | - | 5 | 2 | Cytoplasmic membrane | -0.201 | 64.29 | 8.22 | No | - |
| Fisuc_0841 FSU_1285 | Conserved domain protein | - | - | 2 | Unknown | -0.197 | 27.296 | 6.91 | No | - |
| Fisuc_0002 FSU_0395 | Diaminopimelate dehydrogenase | - | - | 4 | Unknown | -0.17 | 35.752 | 6.76 | No | - |
| Fisuc_0777 FSU_1218 | dTDP-4-dehydrorhamnose 3,5-epimerase | - | - | 2 | Unknown | -0.327 | 21.178 | 5.33 | No | - |
| FSU_2120 | FG-GAP repeat protein | - | - | 2 | Unknown | -0.216 | 121.23 | 5.79 | No | - |
| Fisuc_1632 | FG-GAP repeat protein | - | 2 |  | Non cytoplasmic | -0.193 | 122.994 | 5.93 | Yes (23-24) | - |
| Fisuc_2905 FSU_0167 | FKBP-type peptidyl-prolylcis-trans isomerase domain protein | 4 | 6 | 5 | Periplasm | -0.496 | 48.48 | 8.83 | Yes (21-22) | - |
| Fisuc_2811 FSU_0066 | Glu/Leu/Phe/Val dehydrogenase | - | 5 | 10 | Unknown (multiple location) | -0.235 | 48.622 | 6.86 | No | - |
| Fisuc_0016 FSU_0409 | GTP-binding protein TypA | - | - | 3 | Cytoplasmic membrane | -0.291 | 67.979 | 5.16 | No | - |
| Fisuc_2253 FSU_2798 | Homoserine dehydrogenase | - | - | 5 | Unknown | 0.142 | 45.884 | 5.9 | No | - |
| Fisuc_1750 FSU_2248 | Inner membrane protein oxaA | 3 |  | - | Cytoplasmic membrane | 0.023 | 67.957 | 9.09 | No | - |
| Fisuc_1429 FSU_1897 | LemA family protein | - | 3 | - | Unknown | 0.068 | 20.494 | 7.81 | No | - |
| Fisuc_1456 FSU_1929 | LemA family protein | 2 | 2 | - | Unknown | -0.109 | 22.275 | 8.77 | No | - |
| Fisuc_0202 FSU_0609 | Lipoprotein | 3 |  | - | Non cytoplasmic | -0.501 | 19.76 | 5.01 | Yes (19-20) | [8] |
| FSU_2141 | Mce-like protein | - | 2 | - | Unknown | -0.013 | 35.369 | 5.23 | No | - |
| Fisuc_2965 FSU_0230 | Membrane protein | 17 | 5 | 5 | Non cytoplasmic | -0.207 | 44.92 | 4.64 | Yes (19-20) | - |
| Fisuc_1898 FSU_2404 | Membrane protein | 5 | 8 | 5 | Non cytoplasmic | -0.268 | 48.26 | 4.79 | No | [8] |
| Fisuc_1527 FSU_2009 | Membrane protein | 10 | 7 | 7 | Outer membrane | -0.263 | 78.75 | 5.09 | Yes (25-26) | [8] |
| Fisuc_1528 FSU_2010 | Membrane protein | 6 | 4 | 3 | Non cytoplasmic | -0.195 | 78.195 | 5.64 | Yes ( | [8] |
| Fisuc_0242 FSU_0652 | MORN variant repeat protein | - | - | 2 | Non cytoplasmic | -0.658 | 34.161 | 6.96 | Yes (17-18) | - |
| Fisuc_0392 | Mucin-associated surface protein (MASP) | 2 | - | 3 | Cytoplasmic membrane | 0.085 | 22.813 | 4.75 | Yes (17-18) | - |
| Fisuc_2130 FSU_2665 | NADH dehydrogenase (Quinone) | - | 2 | - | Unknown | -0.065 | 46.835 | 6.02 | No | - |
| Fisuc_2129 FSU_2664 | NADH dehydrogenase (Ubiquinone) 24 kDa subunit | - | 2 |  | Unknown | -0.212 | 37.941 | 6.75 | No | - |
| Fisuc_2126 FSU_2661 | NADH-quinoneoxidoreductase subunit B | - | 2 |  | Cytoplasmic membrane | -283 | 23.97 | 6.62 | No | - |
| Fisuc_2059 FSU_2587 | Oxidoreductase domain protein | - | - | 2 | Periplasm | -0.306 | 45.143 | 8.41 | No | - |
| Fisuc_0062 FSU_0457 | Penicillin-binding protein 1A | - | 5 | 4 | Unknown | -0.394 | 90.22 | 9.33 | No | - |
| Fisuc_1443 FSU_1914 | Peptidase M23 | - |  | 2 | Outer membrane | -0.327 | 30.171 | 9.65 | No | - |
| Fisuc_0871 FSU_1317 | Peptidase M23 | - | 2 | 2 | Non cytoplasmic | -0.201 | 48.044 | 9.71 | No | - |
| Fisuc_0872 FSU_1318 | Peptidyl-prolylcis-trans isomerase | 7 | 8 | 7 | Outer membrane | -0.451 | 29.67 | 7.65 | No | - |
| Fisuc_1756 FSU_2256 | Peptidyl-prolylcis-trans isomerase, FKBP-type | 2 | - | - | Periplasm | -0.115 | 42.84 | 6.36 | Yes (18-19) | - |
| Fisuc_0518 FSU_0941 | Peptidyl-prolylcis-trans isomeraseSurA | 3 | - | - | Periplasm | -0.31 | 48.24 | 6.63 | Yes (19-20) | - |
| Fisuc_0775 FSU_1216 | Polysaccharide biosynthesis/export protein | 6 | 8 | 3 | Out ermembrane | -0.181 | 41.49 | 5.74 | Yes (21-22) | - |
| Fisuc_2762 | PpiC-type peptidyl-prolylcis-trans isomerase | 16 | 18 | 11 | Outer membrane | -0.291 | 71.067 | 5.11 | No | - |
| Fisuc_2974 FSU_0239 | PPIC-type PPIASE domain protein | 10 |  | 3 | Non cytoplasmic | -0.422 | 36.986 | 9.5 | Yes (22-23) | - |
| Fisuc_2068 FSU_2596 | Putative lipoprotein | 8 | 20 | 13 | Unknown | -0.539 | 39.21 | 8.81 | No | - |
| Fisuc_2490 FSU_3058 | Putative lipoprotein | - | 7 | 3 | Unknown | -0.551 | 80.441 | 6.59 | Yes (18-19) | - |
| Fisuc_2493 FSU_3061 | Putative lipoprotein | 17 | 33 | 13 | Cytoplasmic membrane | -0.363 | 70.572 | 6.93 | No | - |
| Fisuc_2572 FSU_3142 | Putative lipoprotein | - | 3 | 3 | Periplasm | -0.205 | 71.611 | 5.67 | Yes (21-22) | - |
| Fisuc_0657 FSU_1088 | Putative lipoprotein | - | 2 |  | Non cytoplasmic | -0.103 | 34.721 | 4.56 | Yes (22-23) | - |
| Fisuc_0752 FSU_1190 | Putative lipoprotein | 2 | - | 2 | Unknown | -0.669 | 22.029 | 4.67 | Yes (21-22) | - |
| Fisuc_1141 FSU_1599 | Putative lipoprotein | - | - | 2 | Unknown | -0.391 | 45.816 | 5.28 | Yes (21-22) | - |
| FSU_2769 | putative lipoprotein | - | 2 |  | unknown | -0.408 | 35.975 | 6.52 | Yes (17-18) |  |
| Fisuc_0220 FSU_0627 | Putative lipoprotein | 4 | 10 | 9 | Unknown | -0.373 | 32.7 | 9.54 | No | - |
| Fisuc_0756 FSU_1194 | Putative lipoprotein | - | - | 2 | Periplasm | -0.128 | 32.275 | 8.54 | Yes (22-23) | - |
| Fisuc_0767 FSU_1207 | Putative lipoprotein | 4 | 5 | 5 | Non cytoplasmic | -0.105 | 37.411 | 4.38 | Yes (24-25) | - |
| Fisuc_2795 FSU_0049 | Putative lipoprotein | 2 | 3 | 3 | Non cytpplasmic | -0.79 | 12.637 | 5.48 | Yes (23-24) | - |
| Fisuc_2897 FSU_0158 | Putative lipoprotein | 2 | - | 4 | Non cytoplasmic | -0.017 | 25.293 | 5.3 | Yes (19-20) | - |
| Fisuc_1021 FSU_1468 | Putative lipoprotein | 4 | 3 | 5 | Non cytoplasmic | -0.41 | 19.71 | 6.72 | Yes (19-20) | - |
| Fisuc_3024 FSU_0289 | Putative lipoprotein | - | - | 2 | Unknown | -0.16 | 31.238 | 4.86 | Yes (5-6) | - |
| Fisuc_1317 FSU_1784 | Putative lipoprotein | - | - | 2 | Non cytoplasmic | -0.371 | 22.441 | 5.18 | Yes (20-21) | - |
| Fisuc_1529 FSU_2011 | Putative lipoprotein | 3 | 3 |  | Non cytoplasmic | -0.279 | 24.182 | 4.8 | Yes (23-24) | - |
| FSU_0522 | Putative lipoprotein | - | 2 |  | Non cytoplasmic | -0.408 | 35.975 | 6.52 | Yes (17-18) | - |
| Fisuc_2739 FSU_3310 | Putative membrane protein | 8 | - | 3 | Cytoplasmic membrane | 0.119 | 115.48 | 6.13 | No | - |
| Fisuc_0081 FSU_0479 | Putative uncharacterized protein | - | 5 | 3 | Unknown | -0.151 | 20.617 | 9.01 | No | - |
| Fisuc_2147 | Putative uncharacterized protein | 8 |  | 8 | Unknown | -0.111 | 24.82 | 6.34 | No | - |
| Fisuc_2269 | Putative uncharacterized protein | 3 | 5 | 5 | Unknown | -0.227 | 26.82 | 10.02 | No | - |
| Fisuc_0382 FSU_0797 | Putative uncharacterized protein | - | 2 | 2 | Cytoplasmic membrane | -0.117 | 37.419 | 5.02 | Yes (18-19) | - |
| Fisuc_0482 FSU_0900 | Putative uncharacterized protein | - | - | 2 | Outer membrane | -0.259 | 54.535 | 5.68 | Yes (21-22) | - |
| Fisuc_0820 FSU_1263 | Putative uncharacterized protein | - | - | 2 | Unknown | -0.295 | 26.781 | 5.36 | No | - |
| Fisuc_0888 FSU_1335 | Putative uncharacterized protein | - | 8 | 3 | Non cytoplasmic | -0.487 | 51.75 | 5.28 | Yes (17-18) | - |
| Fisuc_1013 FSU_1460 | Putative uncharacterized protein | - | 3 | - | Non cytoplasmic | -0.303 | 16.368 | 4.8 | Yes (21-22) | - |
| Fisuc_1203 FSU_1664 | Putative uncharacterized protein | - | 8 | 4 | Unknown | -0.34 | 26.03 | 7.75 | No | - |
| Fisuc_1319 FSU_1786 | Putative uncharacterized protein | 2 | - | 4 | Non cytoplasmic | 0.183 | 27.32 | 5.58 | Yes (19-20) | - |
| Fisuc_1526 FSU_2008 | Putative uncharacterized protein | 4 | 3 | 4 | Non cytoplasmic | -0.338 | 85.239 | 5.5 | Yes (19-20) | - |
| Fisuc_1597 | Putative uncharacterized protein | 2 | 6 | 2 | Non cytoplasmic | 0.144 | 29.528 | 9.01 | Yes (22-23) | - |
| FSU_0247 | Putative uncharacterized protein | - | 2 | - | Outer membrane | -0.23 | 34.983 | 4.8 | No | - |
| FSU_3096 | Putative uncharacterized protein | 2 |  | 3 | Unknown | -0.316 | 34.113 | 5.24 | No | - |
| FSU_0881 | Putative uncharacterized protein | 3 | - | - | Non cytoplasmic | -0.585 | 41.95 | 6.35 | Yes (19-20) | - |
| Fisuc_1875 FSU_2377 | Putative uncharacterized protein | - | - | 4 | Non cytoplasmic | -0.5 | 37.593 | 5.53 | Yes (18-19) | - |
| Fisuc_1954 FSU_2474 | Putative uncharacterized protein | - | 4 | - | Extracellular | -0.26 | 64.462 | 6.63 | No | - |
| Fisuc_2020 FSU_2544 | Putative uncharacterized protein | 2 |  | 3 | Non cytoplasmic | -0.055 | 28.17 | 5.1 | Yes (18-19) | - |
| Fisuc_2072 FSU_2600 | Putative uncharacterized protein | 2 | - | 2 | Outer membrane | -0.117 | 38.859 | 6.05 | Yes (23-24) | - |
| Fisuc_0328 FSU_0743 | Putative uncharacterized protein | - | 2 | 2 | Periplasm | -0.314 | 25.13 | 6.63 | No | - |
| Fisuc_2326 | Putative uncharacterized protein | - | - | 3 | Non cytoplasmic | -0.554 | 23.324 | 8.7 | Yes (34-35) | - |
| Fisuc_0474 FSU_0892 | Putative uncharacterized protein | - | 2 | - | Unknown | 0.11 | 24.669 | 5.58 | No | - |
| Fisuc_2506 FSU_3074 | Putative uncharacterized protein | 3 | - | - | Non cytoplasmic | -0.391 | 34.551 | 4.51 | Yes (21-22) | - |
| Fisuc_2544 FSU_3113 | Putative uncharacterized protein | - | 4 | - | Unknown | -0.026 | 36.422 | 9.55 | No | - |
| Fisuc_2555 FSU_3125 | Putative uncharacterized protein | 2 | 2 |  | Non cytoplasmic | -1.191 | 18.92 | 9.06 | Yes (21-22) | - |
| Fisuc_2763 FSU_0015 | Putative uncharacterized protein | 2 | - | - | Non cytoplasmic | -0.339 | 39.8 | 7.62 | Yes (21-22) | - |
| Fisuc_0861 | Putative uncharacterized protein | - | 2 | - | Unknown | -0.354 | 67.25 | 9.38 | Yes (17-18) | - |
| Fisuc_0866 | Putative uncharacterized protein | - | 3 | 3 | Unknown | -0.534 | 264.045 | 5.11 | No | - |
| Fisuc_2863 | Putative uncharacterized protein | 7 | 10 | 10 | Non cytoplasmic | -0.228 | 56.8 | 5.06 | Yes (17-18) | - |
| Fisuc_3015 FSU_0280 | Putative uncharacterized protein | 3 | 2 | 2 | Non cytoplasmic | -0.229 | 37.819 | 5.41 | Yes (20-21) | - |
| Fisuc_3021 | Putative uncharacterized protein | 3 |  |  | Cytoplasmic membrane | -0.005 | 67.29 | 4.51 | No | - |
| Fisuc_1223 FSU_1684 | Putative uncharacterized protein | - | - | 3 | Non cytoplasmic | 0.167 | 31.235 | 5.21 | Yes (19-20) | - |
| Fisuc_1476 | Putative uncharacterized protein | 4 | - | - | Non cytoplasmic | -0.486 | 43.31 | 8.83 | Yes (20-21) | - |
| Fisuc_1485 FSU_1966 | Putative uncharacterized protein | 4 |  |  | Non cytoplasmic | -0.184 | 21.636 | 8.98 | Yes (21-22) | - |
| FSU_2597 | Putative uncharacterized protein | 2 | - | - | Outer membrane | -0.22 | 41.786 | 4.64 | No | - |
| FSU_2684 | Putative uncharacterized protein | - | 8 | - | Cytoplasmic membrane | -0.1 | 26.421 | 6.93 | No | - |
| FSU_2695 | Putative uncharacterized protein | - | 6 | 2 | Unknown | 0.018 | 27.822 | 8.39 | No | - |
| FSU_2876 | Putative uncharacterized protein | - | 2 | - | Non cytoplasmic | -0.559 | 25.727 | 9.18 | No | - |
| FSU_1004 | Putative uncharacterized protein | 2 | - | - | Unknown | -0.618 | 127.845 | 4.62 | No | - |
| Fisuc_2881 FSU_0139 | Pyruvate-flavodoxinoxidoreductase | 7 | 7 | 17 | Unknown | -0.138 | 129.294 | 7.23 | No | - |
| Fisuc_2732 FSU_3303 | Rhodanese domain protein | - | 5 | 4 | Non cytoplasmic | 0.033 | 15.785 | 8.81 | Yes (20-21) | - |
| Fisuc_2492 FSU_3060 | Succinate dehydrogenase | 2 | 3 | 2 | Cytoplasmic membrane | 0.404 | 31.59 | 9.04 | No | - |
| Fisuc_0663 FSU_1094 | Toluene tolerance family protein | 3 | - | - | Non cytoplasmic | -0.449 | 21.87 | 9.22 | Yes (18-19) | - |
| Fisuc_0033 FSU_0426 | Uncharacterized protein | 2 | 4 | 2 | Unknown | -0.711 | 22.89 | 10.11 | No | - |
| Fisuc_2384 FSU_2941 | UPFO365 protein | 2 | 2 |  | Unknown | 0.265 | 34.8 | 7.76 | No | - |
| Fisuc_0556 FSU_0980 | UPFO365 protein | - | - | 2 | Cytoplasmic membrane | -0.082 | 42.916 | 5.68 | No | - |
